# Simultaneous multi-targeted forensic toxicological screening in biological matrices by MRM-IDA-EPI mode

**DOI:** 10.1007/s00204-024-03806-2

**Published:** 2024-06-25

**Authors:** Martina Franzin, Rebecca Di Lenardo, Rachele Ruoso, Paolo Dossetto, Stefano D’Errico, Riccardo Addobbati

**Affiliations:** 1 Institute for Maternal and Child Health, IRCCS “Burlo Garofolo”, Trieste, Italy; 2 Sciex, Milan, Italy; 3 Department of Medical, Surgical and Health Sciences, University of Trieste, Trieste, Italy; 4 Azienda Sanitaria Universitaria Giuliano Isontina, Trieste, Italy

**Keywords:** Toxicological screening, Biological matrices, Overdose, Forensic cases, Paediatric intoxications

## Abstract

The toxicologist ascertains drug assumptions in case of paediatric intoxications and death for overdose. The analytical approach consists of initially screening and consequently confirming drug positivity. We developed a toxicological screening method and validated its use comparing the results with a LC–MS/MS analysis. The method identifies 751 drugs and metabolites (704 in positive and 47 in negative mode). Chromatographic separation was achieved eluting mobile phase A (10 mM ammonium formate) and B (0.05% formic acid in methanol) in gradient on Kinetex Phenyl-Hexyl (50 × 4.6 mm, 2.6 μm) with 0.7 mL/min flow rate for 11 min. Multiple Reaction Monitoring (MRM) was adopted as survey scan and, after an Information-Dependent Analysis (IDA) (threshold of 30,000 for positive and 1000 cps for negative mode), the Enhanced Product Ion (scan range: 50–700 amu) was triggered. The MS/MS spectrum generated was compared with one of the libraries for identification. Data processing was optimised through creation of rules. Sample preparation, mainly consisting of deproteinization and enzymatic hydrolysis, was set up for different matrices (blood, urine, vitreous humor, synovial fluid, cadaveric tissues and larvae). Cut-off for most analytes resulted in the lowest concentration tested. When the results from the screening and LC–MS/MS analysis were compared, an optimal percentage of agreement (100%) was assessed for all matrices. Method applicability was evaluated on real paediatric intoxications and forensic cases. In conclusion, we proposed a multi-targeted, fast, sensitive and specific MRM-IDA-EPI screening having an extensive use in different toxicological fields.

**Supplementary Information:**

The online version contains supplementary material available at 10.1007/s00204-024-03806-2.

## Introduction

According to the World Drug Report 2023 by the United Nations Office on Drugs and Crime, the incidence of overdoses and intoxications from substances has increased (UNOC 2023). This could be due to the greater abuse of prescription drugs, such as opioids and psychostimulants, and to the increased circulation of new substances of abuse, including the new psychoactive substances (NPS) (Ferrari Júnior et al. 2022; McHugh et al. 2015). These last-mentioned drugs, even if not scheduled, are synthetized to mimic traditional drug effects, thus encouraging addiction (Ferrari Júnior et al. 2022). Addiction has become a serious public health problem and, not only adults, but also adolescents, are affected by this scenario (Kariisa et al. 2019; Mattson et al. 2021; Paul et al. 2018).

Since children have a premature immune system, as well as a natural tendency to explore the environment coming into contact with toxicants, acute poisoning and consequent addressing the paediatric emergency department may occur frequently in this population (Corlade-Andrei et al. 2023).

Therefore, the toxicologist has a fundamental role in ascertaining the assumption of drugs, novel or traditional ones, leading to intoxication or death for overdose. Generally, the analytical approach consists of initially screening the biological material and, once noticed a positivity to substances, in confirming drug presence (Drummer and Gerostamoulos 2014).

To date, the screening methods currently used exploit immunological assays or gas chromatography coupled to mass spectrometry (GC–MS) (McLaughlin et al. 2019; Ramoo et al. 2016). However, immunological assays can sometimes determine false positives, due to cross-reactivity mechanisms with matrix interferents, and generally succeed in determining only traditional drugs of abuse (Franzin et al. 2024; Sotnikov et al. 2021). On the other hand, the GC–MS technique requires a complex pre-analytical phase including the sample derivatization, thus inevitably lengthening the times of analysis (Fiehn 2016). Interestingly, liquid chromatography coupled to mass spectrometry (LC–MS) has become a valid alternative for toxicological screening, thanks to its sensitivity, specificity and short times of analysis (Dresen et al. 2010; Grapp et al. 2018; Mueller et al. 2005). In particular, screening using LC–MS technique exploits high-resolution instruments with an untargeted research or hybrid instruments with a targeted approach (Dresen et al. 2010; Grapp et al. 2018).

Toxicological investigations can be carried out on several biological matrices. Noteworthy, given the presence of specialised personnel on site, urine, as well as blood samples, can be collected at the emergency room in the event of intoxication being ascertained by the doctor (Lee et al. 2021). As deeply described in scientific literature, investigations in blood represent a photograph of the present moment, when venipuncture occurs, whereas urine is indicative of past drug exposure (Corlade-Andrei et al. 2023; Gallardo and Queiroz 2008; Greco et al. 2023).

Instead, postmortem samples are usually collected at autopsy and include blood (peripheral and cardiac) and urine, defined as “traditional matrices” (de Campos et al. 2022; Gallardo and Queiroz 2008). However, these matrices are not always available at autopsy as in the case of high decomposition and fire (Bierly and Labay 2018; de Campos et al. 2022; Gallardo and Queiroz 2008). For this reason, it is important for forensic investigations to use alternative matrices, such as vitreous humor, synovial fluid, cadaveric tissue and cadaveric larvae (Deking et al. 2014; Greco et al. 2023; Groth et al. 2022; Kintz et al. 1990; Savini et al. 2020). Indeed, as necrophagous insects, attracted by the odour produced during decomposition, enter the soft parts of the body and feed on the decomposed tissues containing xenobiotics, they could be indicative of human exposure to drugs of abuse (Groth et al. 2022; Joseph et al. 2011).

In this context, the present work focuses on the development of a toxicological multi-targeted screening method on a hybrid triple quadrupole-ion trap instrument QTRAP 6500 + (Sciex). Beyond the optimization of analytical and statistical method parameters, estimation of the agreement with a LC–MS/MS analysis and applicability to real samples are also presented.

## Materials and methods

### Chemicals, reagents and standards

All chemicals and reagents used were of analytical grade. Methanol (≥ 99.9%) and acetonitrile (≥ 99.9%) were purchased from Merck (Darmstadt, Germany); ultrapure water was obtained from Biosolve Chimie (Dieuze, France). Formic acid (≥ 98%) and ammonium formate (97%) were purchased from Merck (Darmstadt, Germany). The analytical column Kinetex Phenyl-Hexyl (50 × 4.6 mm, 2.6 μm) was obtained from Phenomenex (Torrance, USA). Analytical standards consisting of NPS (Supplementary material—Table S1) were provided by the Italian National Institute of Health. Internal standard mix, consisting of deuterated compounds, was purchased from Chromsystems Instruments and Chemicals GmbH (Munich, Germany).

### Sample collection

Blood (3) and urine (3) samples derived from paediatric cases of suspected intoxication were obtained from the emergency department of IRCCS “Burlo Garofolo” Hospital. Furthermore, postmortem specimens of blood (6), urine (4), vitreous humor (3), synovial fluid (7), cadaveric tissues of liver (4), kidney (4), spleen (3) and cadaveric larvae (5) were obtained from forensic cases with suspected cause of death due to intoxication or overdose and collected during autopsy by forensic pathologists of the University of Trieste and School of Forensic Medicine. All the biological samples were carried to the Advanced Translational Diagnostic Laboratory and stored at – 20 °C until the analysis. Biological samples of this study were left over from routine analyses and their use for analytical validation was approved by IRCCS “Burlo Garofolo” (RC 56/22).

### Sample preparation

For whole blood samples, 5 μL of internal standard (IS) mix was spiked into 90 μL of human whole blood matrix. Samples were extracted using a protein precipitation procedure. Basically, 900 μL of methanol:acetonitrile (50:50, v/v) was added to the sample and vortexed for 1 min. Then, after sonication for 3 min and vortexing for 1 min, samples were centrifuged for 5 min at 14,100 g. The supernatant was transferred into a new tube and dried under nitrogen. The residues were reconstituted with 250 μL of methanol: water (20:80, v/v).

For urine samples, 5 μL of IS and 40 μL of β-glucuronidase enzyme, whose enzymatic hydrolysis efficiency was previously tested by Chromsystems Instruments and Chemicals GmbH (Munich, Germany), were spiked into 50 μL of urine matrix. Afterwards, samples were incubated for 2 h at 45 °C to allow enzymatic deconjugation. At the end of incubation, 100 µL of precipitant reagent was added and, after vortexing, the samples were centrifuged for 5 min at 14,100 g. To 100 µL of supernatant, 150 µL of dilution buffer was added.

For vitreous humor and synovial fluid, 5 μL of IS was spiked into 50 μL of biological matrix. Afterwards, 40 µL of dilution buffer and 100 µL of precipitant reagent were added and, after vortexing, the sample was centrifuged for 5 min at 14,100 g. To 100 µL of supernatant, 150 µL of dilution buffer was added.

For cadaveric tissues of liver and kidney, as well as for cadaveric larvae, 500 mg of matrices was weighed and subsequently 1.5 mL of methanol was added. Specimens were homogenised using the instrument Bead Ruptor Elite (Omni International, Milan, Italy) according to specific grinding protocols previously set up (liver: 2 cycles at 5 m/s for 20 s without pause; kidney: 3 cycles at 4 m/s for 10 s with a pause of 10 s; larvae: 3 cycles at 6 m/s for 15 s with a pause of 5 s). The homogenate was centrifuged for 10 min at 14,100 g. One mL of supernatant was dried under nitrogen and the residues were resuspended in 50 μL of mobile phase A. Then, sample preparation was the same as that for urine matrix.

For cadaveric tissues of spleen, 500 mg of spleen was weighed and subsequently 1.5 mL of methanol was added. Samples were homogenised as described above with the protocol: 2 cycles at 4.5 m/s for 10 s with a pause of 10 s. Then, the homogenate was centrifuged for 10 min at 14,100 g, and 150 µL of dilution buffer was added to 100 µL of supernatant.

### Instrumentation and analytical parameters

Analyses were performed with a HPLC Exion LC 2.0 (Sciex, Milan, Italy) combined with a QTRAP 6500 + system (Sciex, Milan, Italy). To achieve chromatographic separation, gradient elution of mobile phase A (10 mM ammonium formate) and B (0.05% formic acid in methanol) on the analytical reverse-phase column Kinetex Phenyl-Hexyl (50 × 4.6 mm, 2.6 μm), thermostatted at 30 °C, was performed. The mobile phases were replaced every 2 days. A linear gradient (700 μL/min) from 10% B to 98% B in 7.0 min followed by 1.5 min of 98% B and 1.0 min of 10% B was employed. The total chromatographic run-time was 11 min. Sample injection volume is 15 μL. Quality controls at known composition (Supplementary material—Table S2) were injected before starting the analysis of a batch of samples to check the instrumental performance.

The ion source mass spectrometer parameters were as follows: curtain gas, 30 psi; collision gas, high; ion spray voltage, 5400 V for positive mode and − 5400 V for negative mode; capillary temperature, 500 (°C); ion source gas, 55 psi and collision gas, high. Acquisition method setting consists of a survey scan and an Information-Dependent Analysis (IDA) triggered scan. As survey scan, multiple reaction monitoring (MRM) mode with 751 transitions (704 in positive mode and 47 in negative mode) was selected. Compound-dependent parameters for each MRM transitions, such as precursor and product ions, declustering potential (DP), entrance potential (EP), collision energy (CE) and collision cell exit potential (CXP), were reported in Supplementary materials—Tables S3 and S4. Contrary to what was set up for the compounds in negative mode, scheduled MRM of compounds in positive mode was adopted analysing a time window of ± 25 s. Q1 and Q3 were used at unit resolution (0.6–0.8 amu at half height). The IDA intensity threshold was set to 30,000 and 1000 counts per second (cps) for positive and negative mode respectively. The two most intense MRM transitions per cycle exceeding the selected threshold were considered for the dependent enhanced product ion (EPI) scan. For further improvement of the identification of coeluted compounds, the MRM transitions, which triggered the dependent scan twice consecutively, were excluded for EPI scans for 15 s. The EPI scans were performed at a scan range of 50 to 700 amu using the dynamic fill time mode with a scan rate of 10,000 amu/s applying a CES of 35 ± 15 eV. The source and the compound dependent parameters were the same as used for the MRM mode. The MS/MS spectra obtained from the analysis were compared with the ones present in the MS/MS Forensic HR-MS/MS 2.1 library (1820 available spectra) (Sciex, Milan, Italy). Based on the present analytical workflow, the proposed method could be defined a MRM-IDA-EPI screening.

### LC–MS/MS analysis

All the results obtained from the screening test underwent confirmation by detection of specific MRM for each analyte.

### Data processing and statistical analysis

Data processing and analysis were performed using Analyst (version 1.5, Sciex, Milan, Italy) and SCIEX OS (version 2.0, Sciex, Milan, Italy). List of the rules for data processing is reported in Table 1.

**Table 1:** List of apply, custom and combined rules for data processing with indication of the parameters involved and displayed layout

	Parameters	Values	Displayed layout
Apply rules			
Qualitative rule	Reverse fit	> 80%	Box is marked with
		≥ 50%	Box is marked with
		≤ 49%	Box is marked with
Integration acceptance	Integration quality	> 0.6	When at least one rule is not fulfilled, box is marked with **!**
	Asymmetry factor	0.6 < asymmetry factor < 1.6	
	Total width	0.1 < width < 0.85	
Custom rules			
Intensity	Height	> 30,000 cps (positive mode) > 1000 cps (negative mode)	When the rule is not fulfilled, box is coloured in light blue
RT error	Variation of retention time (∆_tr_)	0 min < ∆_tr_ < 0.20 min	When the rule is not fulfilled, box is coloured in red
Library hit	Reverse fit	≤ 49%	The rule reports anomalies and is principally used for the subsequent combined rule
Combined rule			
Intensity + RT error + Library Hit	Intensity	Fulfilled	When intensity and RT error are fulfilled and Library hit is not fulfilled (appropriate intensity, retention time and reverse fit), box is marked with
	RT error	Fulfilled	
	Library hit	Not fulfilled	

Agreement between the screening test and the LC–MS/MS analysis was estimated on the cohort of postmortem specimens calculating the percentage of agreement (confirmed cases/total cases × 100). Furthermore, Cohen’s kappa analysis was also performed as statistical measurement to observe the agreement between the data sets, also taking into account the chance agreement. In particular, kappa (κ) scores between 0.81 and 1 represent perfect agreement, 0.61 and 0.8 substantial agreement, 0.41 and 0.6 moderate agreement and 0.1 and 0.2 slight agreement. Negative values may generally be interpreted as no agreement (McHugh 2012).

## Results

### Method development

#### Chromatographic and spectrometric parameters

**Fig. 1:**
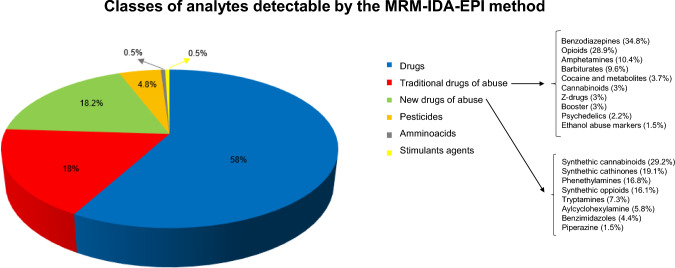
Classes of analytes detectable by the proposed MRM-IDA-EPI method and their percentages on the whole

The proposed MRM-IDA-EPI method allows the detection and identification of 751 analytes, belonging to several classes of drugs of abuse and toxicants (Fig. 1). Interestingly, beyond drugs and traditional illicit substances, a large number of new drugs of abuse, particularly 137 NPS, are found in the toxicological screening.

To check the instrumental performance, in terms of retention times and mass spectral performance, quality controls at known composition of 102 analytes (Supplementary material—Table S2) were injected as system suitability test before starting the sample batch analysis. Furthermore, since an IS mix was added in the sample preparation step, 9 deuterated compounds belonging to the above-mentioned mix were monitored in each sample. Retention times of the deuterated compounds are reported in Table 2 and cover all the chromatographic run.

**Table 2:** Deuterated compounds, present in the IS mix, monitored along the chromatographic run

IS	Retention time (min)
Oximorphone-D3	2.15
Methylone-D3	2.95
6-Monoacetylmorphine-D6	3.14
3,4-Methyl enedioxy-methamphetamine-D3	3.38
Ketamine-D4	3.83
Meperidine-D4	4.37
Cocaethylene-D3	4.72
Promethazine-D6	5.85
Diazepam-D5	6.74

Among the 751 analytes of the proposed screening method, 704 and 47 compounds were ionised in positive and negative mode respectively. Scheduled MRM mode was adopted in positive mode, when a large number of analytes occur. This allows a more targeted and sensitive detection of compounds as well as less interference of matrix components, which do not elute at the retention time of analytes.

#### Assay sensitivity

To estimate the sensitivity of the screening method, 3 standards in urine (102 analytes) and in blood (17 analytes) at known concentrations were analysed and the identification of compounds was evaluated. Supplementary materials—Tables S5 and S6 report the concentration of each compound of interest in each standard and whether they were identified. Interestingly, regarding the standards in urine, only 19 illicit drugs were not identified in the standard at the lowest concentration tested. Furthermore, regarding the standards in blood, 83% of the drugs present were identified in the standard at the lowest concentration tested.

### Agreement between the screening and the LC–MS/MS analysis

Since the proposed MRM-IDA-EPI method was set up as a screening method for toxicological investigations, a comparison with the LC–MS/MS analysis is necessary to test and validate its reliability. Therefore, the agreement between the results obtained from the screening and the LC–MS/MS analyses was estimated for all the biological matrices, which the assay was developed for. Noteworthy, as evidenced in Table 3, the percentage of agreement between the assays was 100% for all biological matrices. As the chance of agreement was also taken into account, Cohen’s kappa score was calculated and described a perfect agreement between the MRM-IDA-EPI screening and LC–MS/MS analyses, where it could be possible to calculate it.

**Table 3:** Indication of the agreement, expressed as percentage of agreement (%) and Cohen’s kappa score (κ), between the proposed MRM-IDA-EPI screening and the LC–MS/MS analysis

Classes of analytes	Agreement (%)	κ
Blood		
Amphetamines	100	NA
Barbiturates	100	NA
Benzodiazepines	100	1
Cocaine and metabolites	100	1
Methadone	100	1
Opioids	100	1
Cannabinoids	100	1
Pregabalin	100	1
Urine		
Amphetamines	100	NA
Barbiturates	100	NA
Benzodiazepines	100	1
Cocaine and metabolites	100	1
Methadone	100	1
Opioids	100	NA
Cannabinoids	100	1
Pregabalin	100	NA
Vitreous humor		
Amphetamines	100	NA
Barbiturates	100	NA
Benzodiazepines	100	1
Cocaine and metabolites	100	1
Methadone	100	1
Opioids	100	1
Cannabinoids	100	NA
Pregabalin	100	1
Synovial fluid		
Amphetamines	100	NA
Barbiturates	100	1
Benzodiazepines	100	1
Cocaine and metabolites	100	1
Methadone	100	1
Opioids	100	1
Cannabinoids	100	NA
Pregabalin	100	1
Cadaveric liver		
Amphetamines	100	NA
Barbiturates	100	NA
Benzodiazepines	100	1
Cocaine and metabolites	100	1
Methadone	100	NA
Opioids	100	NA
Cannabinoids	100	1
Pregabalin	100	NA
Cadaveric kidney		
Amphetamines	100	NA
Barbiturates	100	NA
Benzodiazepines	100	1
Cocaine and metabolites	100	1
Methadone	100	NA
Opioids	100	NA
Cannabinoids	100	1
Pregabalin	100	NA
Cadaveric spleen		
Amphetamines	100	NA
Barbiturates	100	NA
Benzodiazepines	100	1
Cocaine and metabolites	100	NA
Methadone	100	1
Opioids	100	NA
Cannabinoids	100	NA
Pregabalin	100	NA
Cadaveric larvae		
Amphetamines	100	NA
Barbiturates	100	NA
Benzodiazepines	100	1
Cocaine and metabolites	100	1
Methadone	100	1
Opioids	100	1
Cannabinoids	100	NA
Pregabalin	100	1

### Method applicability

#### Emergency department paediatric intoxications

After parents addressed the emergency department for suspected intoxication of the antidepressant bupropion, blood and urine samples of a newborn were collected and screened. Bupropion and its metabolite hydroxy bupropion, as well as the drugs administered in the emergency department (the benzodiazepines midazolam and lorazepam), were identified in all the biological matrices available.

After showing up at the emergency department with hallucinations, whole blood and urine samples from 2 paediatric patients were screened. Whilst blood resulted to be negative to all the substances present in the MRM-IDA-EPI method, pregabalin and gabapentin were identified in the urines of these paediatric patients, ascertaining a previous exposure to these anticonvulsants and probably causing the above-mentioned adverse effects.

#### Forensic cases

Postmortem specimens obtained from 21 forensic cases with suspected death for overdose were analysed. Among the forensic cases screened, 17 (80%) resulted positive to drug assumption. In detail, the most identified illicit drugs and metabolites evidenced a prevalent consumption of benzodiazepines (52%) and methadone (38%), followed by cocaine (33%) and opioids (19%). Quetiapine (14%), 11-nor-9-tetrahydrocannabinol-9-carboxylic acid (THC-COOH) and zolpidem (10%), barbiturates and pregabalin (5%) were detected to a lesser extent in postmortem samples.

Interestingly, beyond the analysis of traditional biological matrices, commonly performed routinely in toxicologic laboratory, our MRM-IDA-EPI method is suitable for the screening of unconventional matrices, sometimes the only available in case of severe decomposition and fire. In detail, Table 4 is representative of the reliability and concordance of the results obtained from different biological matrices, traditional or not, belonging to the same forensic case. The presentation of all the analysed forensic cases resulted positive is also reported in Supplementary materials—Table S6.

**Table 4:** Presentation of 3 forensic cases including the results of MRM-IDA-EPI screening and LC–MS/MS analysis

Biological matrix	MRM-IDA-EPI screening positivity	LC–MS/MS analysis
First forensic case		
Peripheric blood	7-Aminoclonazepam Cocaine Benzoylecgonine (BEG) Methadone 2-Ethylidene-1,5-dimethyl-3,3-diphenylpyrrolidine (EDDP)	408.14 μg/L 199.77 μg/L 1562.65 μg/L 338.37 μg/L 296.15 μg/L
Urine	7-Aminoclonazepam Alprazolam Hydroxy-alprazolam Cocaine BEG Cocaethylene Methadone EDDP THC-COOH	1309.46 μg/L 11.10 μg/L 51.58 μg/L 2168.65 μg/L 10,479.35 μg/L 15.47 μg/L 1965.44 μg/L 3452.52 μg/L 20.68 μg/L
Synovial Fluid	7-Aminoclonazepam Cocaine BEG Methadone EDDP	170.34 μg/L 5.8 μg/L 705.03 μg/L 383.32 μg/L 24.16 μg/L
Vitreous humor	7-Aminoclonazepam Cocaine BEG Methadone EDDP	112.53 μg/L 51.83 μg/L 626.96 μg/L 212.29 μg/L 20.71 μg/L
Second forensic case		
Cadaveric liver	Diazepam Nordiazepam Oxazepam Temazepam	235.0 ng/g 174.0 ng/g 12.0 ng/g 9.0 ng/g
Cadaveric kidney	Diazepam Nordiazepam Oxazepam Temazepam	89.0 ng/g 26.0 ng/g 5 ng/g 14 ng/g
Cadaveric larvae	Diazepam Nordiazepam Oxazepam Temazepam	3.0 ng/g 6.0 ng/g 30.0 ng/g < LOQ
Third forensic case		
Cadaveric spleen	7-aminoclonazepam Quetiapine Methadone EDDP	706.0 ng/g 3693.0 ng/g 27,699.0 ng/g 1990.0 ng/g
Cadaveric larvae	7-aminoclonazepam Quetiapine BEG Methadone EDDP	4.0 ng/g 13.0 ng/g 5.0 ng/g 127.0 ng/g 101.0 ng/g

## Discussion and conclusion

Overdoses and intoxications have become a global burden and have increasingly raised leading to fatal events (Corlade-Andrei et al. 2023; UNOC 2023). In this context, several molecules could be the responsible for addiction and intoxications and the circulation of new drugs, particularly NPS, that mimic the traditional illicit effects without being scheduled, occurs.

In this context, the development of fast, sensitive and specific methods to screen a large number of drugs of abuse and toxicants results fundamental to understand the forensic cases as well to ascertain paediatric intoxications.

Noteworthy, the proposed MRM-IDA-EPI method is a multi-targeted screening method without exploiting high-resolution instrumentations. In detail, till 751 analytes could be identified using liquid chromatography coupled with QTRAP technology of the manufacturer Sciex. Another previous work focussed on screening using an untargeted approach through high-resolution instrumentations (Pierre Negri 2019). To the authors’ knowledge, this is the first time such a wide number of analytes could be screened for toxicological investigations with the QTRAP technology (Dresen et al. 2010; Merone et al. 2022). Moreover, the instrument used in the proposed MRM-IDA-EPI method allowed a higher sensitivity in comparison with the previous studies by Dresen and Merone (Dresen et al. 2010; Merone et al. 2022). Interestingly, after evaluating compounds identification in several concentration levels and in two different matrices, the high sensitivity of the proposed method was underlined as the lowest concentration tested, which resulted in the cut-off for most analytes.

Different from screening by immunoassays, the proposed method is specific in avoiding false positivity for cross-reactivity mechanisms (Franzin et al. 2024; Moody et al. 2022; Nieddu et al. 2022; Wang et al. 2014). Indeed, compounds related to the biological matrix, such as sympathomimetic and putrefactive amines, that usually cross react in immunoassays, do not interfere in the present screening (Bonicelli et al. 2022; Broussard 2008; McLaughlin et al. 2019). As a proof of this fact, the agreement between the LC–MS/MS analysis and the proposed screening method is optimal, indicating no false results.

Contrary to what was reported in previous scientific literature, the MRM-IDA-EPI method was set up on several biological matrices evidencing its extensive use in several toxicological fields (Dresen et al. 2010; Merone et al. 2022; Pierre Negri 2019). Beyond traditional matrices, also unconventional matrices, whose employment has been incremented in peculiar cases, were tested. This could be another advantage in comparison to immunoassays, which are usually validated on traditional matrices (Greco et al. 2023; Molina and Dimaio 2005; Rasanen et al. 2000).

Moreover, the sample preparation was optimised to be fast and allows rapid response times. It consists mainly of protein precipitation through the use of organic solvents as other works did before (Pierre Negri 2019). Also, the sample preparation does not differ based on the concentrations supposed in the samples, as Dresen described previously (Dresen et al. 2010).

Generally, contrary to blood, vitreous humor and synovial fluid, the other biological matrices, are indicative of previous drug exposure and accumulate both in the parent drug and the corresponding metabolite derived from hepatic metabolism (de Campos et al. 2022; Hadland and Levy 2016; Vanbinst et al. 2002). Therefore, a further step of preparation consisting of enzymatic hydrolysis to convert the metabolite in the deconjugated molecule is needed for identification. Nonetheless, the sample preparation could be also faster, avoiding this step, as we included some glucoronates compounds, mainly belonging to opiates class, in the analytes list as previously done (Dresen et al. 2010).

Interestingly, since the sample preparation for biological matrices is composed of several steps, an IS mix was added and the 9 deuterated compounds present in it were monitored in every chromatographic run. In addition, another system suitability test adopted was the injection of a quality control with till 102 known substances. To the authors’ knowledge, previous works limited the instrumental check to a lower number of molecules injected before the samples batch (Dresen et al. 2010; Merone et al. 2022).

Interestingly, the applicability of the present screening method was evaluated on different kinds of samples. Regarding paediatric samples, results obtained by MRM-IDA-EPI screening validate the emergency department clinicians’ hypothesis of intoxications. Furthermore, regarding the presentation of some representative forensic cases, there is an evident concordance between the parent drugs and the metabolites identified in several matrices belonging to the same individual. When metabolites were found in blood as well as in vitreous humor and synovial fluid, they were products of hydrolysis by enzymes present in the circulatory stream (Matsubara et al. 1984; Meyer et al. 2015; Stewart et al. 1979).

Interestingly, the screening method can potentially identify NPS, that probably immunoassays could not identify for unavailability of antibodies specific for the compounds recently synthesised (Moeller et al. 2017).

Our work proposed a fast, sensitive and specific multi-targeted tool for forensic toxicology screening in biological matrices, obtained from paediatric patients or forensic cases with suspected intoxications and overdoses respectively. It could be exploited also for investigations of non-biological materials derived from seizure, taking into account the wide number of analytes potentially identified.

## Supplementary Information

Below is the link to the electronic supplementary material.

Supplementary file1 (DOCX 205 KB)

## Data Availability

Data are available from the corresponding author on reasonable request.
