# Supplementary material for: Simultaneous multi-targeted forensic toxicological screening in biological matrices by MRM-IDA-EPI mode
Source: Arch Toxicol. 2024 Jun 25;98(10):3231–40. doi: 10.1007/s00204-024-03806-2 (PMC11402837; doi:10.1007/s00204-024-03806-2)
Supplement: Supplementary file 1 — Supplementary file1 (DOCX 205 KB) [file 204_2024_3806_MOESM1_ESM.docx]

**Table S1.** Analytical standards containing new psychoactive substances (NPS).

| **BOX C** | **BOX D** | **BOX E** |
| --- | --- | --- |
| 2-fluoro Deschloroketamine | 5-Hydroxytryptophan | 3-Methylmethcathinone (hydrochloride) |
| Alpha-PPP  (α-Pyrrolidinopropiophenone) | 5-APB (hydrochloride) | 4-fluoro-MDMB-BUTICA |
| 2-methyl AP-237 (hydrochloride) | 5-Chloro AB-PINACA | 5-fluoro CUMYL-PICA |
| 3,4-MD-alpha-PHP | 5-chloro THJ 018 | 5-fluoro EDMB-PICA |
| 3-methoxy-PCE | 5-fluoro NNEI 2-naphthyl isomer | 5-Fluoro EMB-PICA |
| 5-fluoro CUMYL-P7AICA | 5-fluoro-APINACA (5F-AKB48) | 5-MMPA (hydrochloride) |
| 5-fluoro CUMYL-PeGACLONE | 5-fluoroAPP-PICA | ADB-4en-PINACA |
| 5-fluoro MDMB-PICA | 5-fluoroAPP-PINACA | Brorphine (hydrochloride) |
| AP-237 (hydrochloride) | 5-fluoroCumyl-PINACA | Butonitazene |
| Bentazepam | 6-APB (hydrochloride) | Etodesnitazene (cytrate) |
| Cinazepam | 6-MAPB (hydrochloride) | Flunitazene (hydrochloride) |
| Clonazolam | AB-CHMINACA | MDMB-4en-PICA |
| Deschloro-N-ethyl-Ketamine | AB-FUBINACA | MDMB-4en-PINACA |
| Diclazepam | Acetyl Fentanyl (hydrochloride) | MDMB-4en-PINACA Butanoic COOH |
| Ethylone (hydrochloride) | ADB-FUBINACA | Methoxpropamine (hydrochloride) |
| Etizolam | Alfentanil (hydrochloride) | Metodesnitazene (hydrochloride) |
| Euthylone | APP-FUBINACA | Metonitazene (hydrochloride) |
| Fentanyl | Butyryl fentanyl (hydrochloride) | N-Pyrrolidino Etonitazene |
| Flualprazolam | Butyryl Norfentanyl (hydrochloride) |  |
| Furanyl fentanyl (hydrochloride) | Carfentanyl |  |
| Isobutyryl fentanyl (hydrochloride) | CUMYL-PeGACLONE |  |
| Isotonitazene | Cyclopropyl fentanyl (hydrochloride) |  |
| Methoxyacetyl fentanyl (hydrochloride) | Furanyl Norfentanyl (hydrochloride) |  |
| N-Ethyl pentylone (hydrochloride) | MDMB-CHIMICA |  |
| Ocfentanyl (hydrochloride) | Metoxy acetil norfentanyl (hydrochloride) |  |
| Para-fluoro-Furanyl fentanyl (hydrochloride) | MMB-2201 |  |
| UR-144 | Ritalinic Acid |  |
|  | Trans-3-metil-Norfentanyl |  |

**Table S2.** Concentration of quality control set, used to check instrumental performance at the beginning of the samples batch.

| **Substance** | **Control level I**  Concentration (μg/L) | **Control level II**  Concentration (μg/L) | **Control level III**  Concentration (μg/L) |
| --- | --- | --- | --- |
| **Amphetamines** |  |  |  |
| Amphetamine | 53.9 | 157 | 513 |
| BDB | 53.8 | 154 | 501 |
| Butylone | 54.5 | 157 | 551 |
| 2C-B | 48.1 | 141 | 539 |
| 2C-I | 53.3 | 153 | 461 |
| Cathinone | 56.4 | 166 | 559 |
| MBDB | 53.2 | 158 | 542 |
| MDA | 52.8 | 160 | 614 |
| MDEA | 52.4 | 153 | 565 |
| MDMA | 48.1 | 140 | 515 |
| MDPV | 53.2 | 158 | 554 |
| Mephedrone | 52.3 | 154 | 556 |
| Methamphetamine | 53.5 | 156 | 545 |
| Methaqualone | 51.9 | 147 | 509 |
| Methylone | 48.5 | 143 | 515 |
| Methylphenidate | 54.4 | 157 | 519 |
| PMA | 51.3 | 141 | 511 |
| Ritalinic Acid | 49.9 | 140 | 471 |
| **Barbiturates** |  |  |  |
| Allobarbital | 155 | 446 | 1581 |
| Amobarbital | 148 | 411 | 1377 |
| Barbital | 159 | 493 | 1881 |
| Butalbital | 151 | 446 | 1740 |
| Hexobarbital | 163 | 461 | 1503 |
| Pentobarbital | 150 | 464 | 1742 |
| Phenobarbital | 153 | 462 | 1754 |
| Secbutabarbital | 158 | 442 | 1440 |
| Secobarbital | 147 | 443 | 1691 |
| **Benzodiazepines** |  |  |  |
| Alprazolam | 52.4 | 154 | 525 |
| 7-aminoclonazepam | 46.9 | 141 | 493 |
| 7-aminoflunitrazepam | 47.3 | 145 | 526 |
| Bromazepam | 52.4 | 150 | 529 |
| Brotizolam | 55.9 | 165 | 586 |
| Chlordiazepoxide | 36.2 | 148 | 539 |
| Clobazam | 51.2 | 141 | 493 |
| Clonazepam | 50 | 146 | 506 |
| Demoxepam | 48.7 | 165 | 597 |
| Desalkylflurazepam | 52.5 | 151 | 540 |
| Desmethylflunitrazepam | 55.9 | 161 | 537 |
| Diazepam | 53.6 | 158 | 557 |
| Estazolam | 56.4 | 154 | 540 |
| Flunitrazepam | 51.4 | 152 | 560 |
| Flurazepam | 51.1 | 147 | 534 |
| A-hydroxyalprazolam | 50.7 | 149 | 512 |
| A-hydroxymidazolam | 53 | 154 | 504 |
| A-hydroxytriazolam | 51.5 | 153 | 509 |
| Lorazepam | 51.4 | 156 | 577 |
| Lormetazepam | 55.8 | 167 | 595 |
| Medazepam | 55.5 | 161 | 548 |
| Midazolam | 48.7 | 139 | 479 |
| Nitrazepam | 46.6 | 148 | 570 |
| Norclobazam | 55.4 | 164 | 568 |
| Nordiazepam | 56.4 | 159 | 575 |
| Oxazepam | 51.6 | 148 | 516 |
| Prazepam | 56.3 | 160 | 545 |
| Temazepam | 52.5 | 164 | 639 |
| Triazolam | 53 | 157 | 585 |
| **Booster** |  |  |  |
| Gabapentin | 52.3 | 160 | 602 |
| Pregabalin | 51 | 153 | 544 |
| Promethazine | 50.7 | 155 | 594 |
| Quetiapine | 52.9 | 154 | 546 |
| **Cocaine** |  |  |  |
| Benzoylecgonine | 30.8 | 91.4 | 336 |
| Cocaethylene | 33 | 96.6 | 346 |
| Cocaine | 32.7 | 95.1 | 347 |
| Norcocaine | 30.2 | 88.5 | 319 |
| **Cannabinoids** |  |  |  |
| 11-nor-9-carboxy-D9-THC | 12.6 | 36.6 | 135 |
| **Opiates/Opioids** |  |  |  |
| Buprenorphine | 1.03 | 2.98 | 11.2 |
| Codeine | 23.4 | 69.7 | 277 |
| Dihydrocodeine | 23.8 | 67.6 | 227 |
| EDDP | 50.6 | 147 | 526 |
| Fentanyl | 9.65 | 27.9 | 95.8 |
| Hydrocodone | 28 | 84.4 | 312 |
| Hydromorphone | 11 | 32.9 | 122 |
| Meconin | 49.9 | 153 | 521 |
| Meperidine | 26.8 | 80.1 | 282 |
| Methadone | 56.4 | 165 | 577 |
| 6-monoacetylmorphine | 5.47 | 16.8 | 64.1 |
| Morphine | 26.5 | 78.2 | 281 |
| Naloxone | 55.4 | 158 | 513 |
| Naltrexone | 53.4 | 153 | 516 |
| Norbuprenorphine | 1.12 | 3.38 | 12.7 |
| Norcodeine | 28 | 82.6 | 309 |
| Norfentanyl | 11.2 | 33.4 | 129 |
| Normeperidine | 28.5 | 81.2 | 287 |
| Nortilidine | 53.7 | 155 | 563 |
| O-desmethyltramadol | 53.7 | 157 | 544 |
| Oxycodone | 52.1 | 149 | 561 |
| Oxymorphone | 26.2 | 76.6 | 275 |
| Papaverine | 56.7 | 169 | 588 |
| Propoxyphene | 52 | 153 | 545 |
| Sufentanil | 53.7 | 155 | 542 |
| Tapentadol | 25 | 73.6 | 269 |
| Thebaine | 50.1 | 149 | 550 |
| Tilidine | 52 | 153 | 551 |
| Tramadol | 52.2 | 153 | 531 |
| **Z-Drugs** |  |  |  |
| Zalepon | 51.5 | 156 | 580 |
| Zolpidem | 55.8 | 164 | 547 |
| Zopiclone | 52.8 | 158 | 565 |
| **Others** |  |  |  |
| Ketamine | 53.1 | 160 | 601 |
| LSD | 0.832 | 2.59 | 9.84 |
| Mescaline | 10.9 | 31 | 95.4 |
| Norketamine | 56.4 | 168.00 | 621.00 |
| PCP | 10.6 | 31.1 | 119 |

**Table S3.** Compound dependent parameters for positive MRMs. t_R_ retention time; DP declustering potential; EP entrance potential; CE collision energy; CXP collision cell exit potential.

| **Opiates/Opioids** | **Q1** | **Q3** | **t_R_ (min)** | **DP (V)** | **EP (V)** | **CE (V)** | **CXP**  **(V)** |
| --- | --- | --- | --- | --- | --- | --- | --- |
| 6-MAM | 328.2 | 165.1 | 3.14 | 100 | 10 | 48 | 8 |
| 6-MAM IS | 334.4 | 165.4 | 3.14 | 140 | 10 | 53 | 10 |
| 6-Monocetylcodeine | 342.2 | 225.1 | 4.36 | 130 | 10 | 35 | 10 |
| Buprenorphine | 468.3 | 55 | 5.35 | 120 | 10 | 85 | 10 |
| Buprenorphine glucuronide | 644.3 | 468.4 | 4.46 | 20 | 10 | 55 | 10 |
| Codeine | 300.1 | 215.1 | 2.89 | 100 | 10 | 35 | 10 |
| Codeine glucuronide | 476.3 | 300.3 | 2.55 | 80 | 10 | 44 | 12 |
| Desomorphine | 272.1 | 215.1 | 3.16 | 80 | 10 | 40 | 12 |
| Dihydrocodeine | 302.1 | 199.1 | 2.82 | 95 | 10 | 44 | 10 |
| EDDP | 278.1 | 234.1 | 5.31 | 80 | 10 | 50 | 12 |
| Heroin | 370.2 | 165 | 4.15 | 80 | 10 | 50 | 12 |
| Hydrocodone | 300.1 | 199 | 2.89 | 100 | 10 | 50 | 12 |
| Hydromorphone | 286.1 | 185 | 2.02 | 100 | 10 | 39 | 10 |
| Hydromorphone glucuronide | 462.3 | 286.2 | 1.08 | 50 | 10 | 41 | 12 |
| Meconin | 195.1 | 77 | 4.47 | 50 | 10 | 48 | 10 |
| Meperidine | 248.1 | 220.1 | 4.37 | 86 | 10 | 28 | 10 |
| Meperidine-IS | 252.1 | 178.1 | 4.37 | 90 | 10 | 29 | 10 |
| Methadone | 310.1 | 265 | 5.91 | 80 | 10 | 25 | 12 |
| Morphine | 286 | 201 | 2.02 | 90 | 10 | 35 | 12 |
| Morphine glucuronide | 462.2 | 286.2 | 1.08 | 60 | 10 | 35 | 15 |
| N-desmethyltapentadol Garbage | 209.1 | 108.1 | 4.24 | 62 | 10 | 30 | 10 |
| Naloxone | 328.1 | 212.1 | 2.82 | 90 | 10 | 49 | 12 |
| Naltrexone | 342.1 | 267.2 | 3.05 | 86 | 10 | 39 | 12 |
| Norbuprenorphine | 414.3 | 55 | 4.86 | 120 | 10 | 90 | 12 |
| Norbuprenorphine glucuronide | 590 | 414 | 3.76 | 60 | 10 | 50 | 12 |
| Norcodeine | 286.1 | 152 | 2.75 | 80 | 10 | 75 | 10 |
| Norhydrocodone | 286.1 | 199 | 3.17 | 70 | 10 | 39 | 11 |
| Normeperidine | 234.1 | 160 | 4.4 | 45 | 10 | 30 | 14 |
| Noroxycodone | 302.1 | 187.1 | 3.08 | 40 | 10 | 33 | 10 |
| Norpropoxyphene | 308.1 | 100 | 5.71 | 50 | 10 | 18 | 10 |
| Nortilidine | 261.1 | 155.1 | 4.47 | 30 | 10 | 24 | 10 |
| O-Desmethyltramadol | 250.1 | 58.1 | 3.12 | 55 | 10 | 97 | 12 |
| Oxycodone | 316.1 | 241.1 | 3.12 | 75 | 10 | 38 | 10 |
| Oxymorphone | 302 | 227.1 | 2.15 | 86 | 10 | 39 | 12 |
| Oxymorphone glucuronide | 478.3 | 284.2 | 1.05 | 80 | 10 | 40 | 12 |
| Oxymorphone-IS | 305.2 | 230.1 | 2.15 | 110 | 10 | 37 | 10 |
| Papaverine | 340.2 | 202.2 | 4.84 | 60 | 10 | 35 | 12 |
| Tapentadol | 222.1 | 107 | 4.15 | 100 | 10 | 40 | 10 |
| Tebaine | 312.2 | 58.0 | 4.36 | 30 | 10 | 45 | 10 |
| Tilidine | 275.1 | 155.1 | 4.47 | 40 | 10 | 27 | 10 |
| Tramadol | 264.1 | 58.1 | 4.04 | 66 | 10 | 103 | 8 |
| **Cannabinoids** |  |  |  |  |  |  |  |
| CBD | 315 | 193 | 7.39 | 50 | 10 | 27 | 10 |
| CBN | 311.2 | 223 | 7.68 | 50 | 10 | 30 | 10 |
| **Cocaine** |  |  |  |  |  |  |  |
| Benzoylecgonine | 290.1 | 168 | 4.17 | 80 | 10 | 37 | 12 |
| Cocaethylene | 318.2 | 196.1 | 4.72 | 61 | 10 | 25 | 10 |
| Cocaethylene-IS | 321.2 | 199.1 | 4.72 | 70 | 10 | 27 | 10 |
| Cocaine | 304.1 | 182.3 | 4.33 | 61 | 10 | 25 | 10 |
| Ecgonine Methyl Ester | 200.1 | 182.1 | 0.85 | 45 | 10 | 23 | 10 |
| Norcocaine | 290.2 | 168 | 4.52 | 50 | 10 | 21 | 10 |
| **Amphetamines** |  |  |  |  |  |  |  |
| 2-Fluoromethamphetamine | 168.1 | 108.9 | 3.38 | 60 | 10 | 25 | 12 |
| 4-Fluoromethamphetamine | 168.1 | 109.1 | 3.38 | 46 | 10 | 23 | 12 |
| Amphetamine | 136 | 91 | 2.9 | 40 | 10 | 40 | 10 |
| DOM (2,5-dimetossi-4-metilamfetamina) | 210.2 | 193.1 | 4.49 | 80 | 10 | 18 | 10 |
| Etilamfetamine (N-Ethylamphetamine) | 164.1 | 91 | 3.44 | 80 | 10 | 29 | 10 |
| Fenetylline | 342.2 | 91 | 4.49 | 60 | 10 | 50 | 12 |
| MDA | 180.1 | 105 | 3.18 | 40 | 10 | 30 | 10 |
| MDEA | 208.1 | 163.1 | 3.66 | 100 | 10 | 20 | 12 |
| MDMA | 194 | 163 | 3.38 | 100 | 10 | 20 | 10 |
| MDMA-IS | 197.1 | 163.1 | 3.38 | 30 | 10 | 17 | 10 |
| Methamphetamine | 150 | 119 | 3.14 | 60 | 10 | 15 | 8 |
| Methaqualone | 251.1 | 91.1 | 6.01 | 60 | 10 | 50 | 12 |
| Methiopropamine | 156.1 | 97.1 | 2.66 | 50 | 5 | 31 | 10 |
| PMA | 166.1 | 149.1 | 3.2 | 80 | 10 | 14 | 10 |
| Ritalinic Acid | 220.1 | 84 | 3.78 | 60 | 10 | 60 | 12 |
| **Benzodiazepines** |  |  |  |  |  |  |  |
| 2-Amino-5-chlorobenzophenone | 232.1 | 154.1 | 6.82 | 60 | 10 | 20 | 10 |
| 2-Amino-5-nitrobenzophenone | 243.1 | 165.1 | 6.46 | 60 | 10 | 20 | 10 |
| 7-Aminoclonazepam | 286.1 | 121.1 | 4.39 | 80 | 10 | 37 | 8 |
| 7-Aminodesmethylflunitrazepam | 270.1 | 121.1 | 4.1 | 60 | 10 | 35 | 15 |
| 7-Aminoflunitrazepam | 284.1 | 135.1 | 4.74 | 71 | 10 | 37 | 10 |
| Alpha-Hydroxyalprazolam | 325.1 | 297.1 | 6.12 | 60 | 10 | 40 | 15 |
| Alpha-hydroxymidazolam | 342.1 | 203.1 | 6.19 | 60 | 10 | 36 | 10 |
| Alpha-hydroxytriazolam | 359.1 | 331.1 | 5.89 | 90 | 10 | 37 | 12 |
| Alprazolam | 309.1 | 281.1 | 6.28 | 80 | 10 | 35 | 10 |
| Bentazepam | 297.1 | 166.2 | 6.5 | 91 | 10 | 39 | 12 |
| Bromazepam | 316.1 | 182.2 | 5.63 | 76 | 10 | 37 | 10 |
| Brotizolam | 393.2 | 282.1 | 6.42 | 100 | 10 | 43 | 10 |
| Chlordiazepoxide | 300 | 227.1 | 6.34 | 56 | 10 | 21 | 10 |
| Cinazepam | 467.1 | 349.1 | 6.4 | 60 | 10 | 20 | 12 |
| Clobazam | 301.1 | 259.2 | 6.18 | 86 | 10 | 29 | 10 |
| Clobenzepam | 316.1 | 271.1 | 4.96 | 60 | 10 | 20 | 10 |
| Clonazepam | 316.1 | 270.2 | 6.03 | 61 | 10 | 35 | 10 |
| Clonazolam | 354 | 308.1 | 5.94 | 97 | 10 | 38 | 12 |
| Clozapine | 326.9 | 270.1 | 5.49 | 60 | 10 | 31 | 10 |
| Delorazepam | 305.2 | 140.2 | 6.38 | 60 | 5,5 | 35 | 15 |
| Demoxepam | 287.1 | 180.1 | 5.88 | 110 | 10 | 31 | 10 |
| Desalkylflurazepam | 289.1 | 140 | 6.19 | 90 | 10 | 40 | 10 |
| Desmethylclobazam | 287.1 | 245.1 | 5.93 | 60 | 10 | 35 | 10 |
| Diazepam | 285.1 | 193 | 6.74 | 80 | 10 | 40 | 10 |
| Diazepam-IS | 290.1 | 154.1 | 6.74 | 100 | 10 | 37 | 10 |
| Diclazepam | 319 | 154.1 | 6.63 | 100 | 10 | 39 | 12 |
| Estazolam | 295.1 | 267.2 | 6.11 | 81 | 10 | 31 | 10 |
| Etizolam | 343.1 | 314.2 | 6.41 | 88 | 10 | 36 | 12 |
| Flualprazolam | 327.1 | 292.2 | 6.12 | 123 | 10 | 36 | 12 |
| Flunitrazepam | 314.1 | 268.2 | 6.21 | 71 | 10 | 39 | 10 |
| Flurazepam | 389.2 | 315.1 | 5.42 | 90 | 10 | 35 | 10 |
| Lorazepam | 321.1 | 275.1 | 6.03 | 80 | 10 | 40 | 10 |
| Lorazepam glucuronide | 497.1 | 275.1 | 5.19 | 90 | 10 | 53 | 12 |
| Lormetazepam | 336.1 | 290.1 | 6.40 | 50 | 10 | 35 | 10 |
| Medazepam | 271.1 | 91 | 6.06 | 60 | 10 | 50 | 12 |
| Midazolam | 326.1 | 291.1 | 6.04 | 101 | 10 | 45 | 12 |
| Nefopam | 254.2 | 181.2 | 4.8 | 60 | 10 | 20 | 12 |
| Nitrazepam | 282.1 | 236.1 | 6.19 | 100 | 10 | 33 | 10 |
| Norclozapine | 313 | 270.2 | 5.46 | 110 | 10 | 36 | 10 |
| Nordiazepam | 271.1 | 140.1 | 6.49 | 71 | 10 | 37 | 10 |
| Norflunitrazepam | 300.1 | 254.1 | 5.88 | 60 | 10 | 30 | 12 |
| Oxazepam | 287.1 | 163.1 | 6.16 | 76 | 10 | 31 | 12 |
| Prazepam | 326.2 | 272.1 | 7.20 | 60 | 10 | 31 | 10 |
| Temazepam | 301.1 | 255.1 | 6.42 | 70 | 10 | 50 | 10 |
| Temazepam glucuronide | 477.1 | 301.2 | 5.58 | 90 | 10 | 23 | 12 |
| Triazolam | 343 | 308.2 | 6.08 | 90 | 10 | 37 | 10 |
| Zolazepam | 287 | 243.1 | 4.88 | 90 | 10 | 25 | 12 |
| **Z-Drugs** |  |  |  |  |  |  |  |
| Zaleplon | 306.2 | 236.3 | 5.79 | 56 | 10 | 35 | 12 |
| Zolpidem | 308.1 | 235.1 | 4.79 | 85 | 10 | 60 | 12 |
| Zolpidem 6-carboxylic acid | 338.2 | 265.2 | 4.88 | 80 | 10 | 52 | 10 |
| Zopiclone | 389.1 | 245.2 | 4.65 | 90 | 10 | 25 | 10 |
| **Barbiturates** |  |  |  |  |  |  |  |
| Primidone | 219.1 | 119 | 4.08 | 50 | 10 | 23 | 11 |
| **Booster** |  |  |  |  |  |  |  |
| Gabapentin | 172.1 | 137 | 2.29 | 50 | 10 | 20 | 10 |
| Pregabalin | 160.1 | 55.1 | 2.19 | 58 | 10 | 31 | 10 |
| Promethazine | 285.1 | 86 | 5.85 | 61 | 10 | 29 | 10 |
| Promethazine-IS | 291.1 | 92 | 5.85 | 55 | 10 | 21 | 10 |
| Quetiapine | 384.2 | 253.1 | 5.6 | 70 | 10 | 35 | 10 |
| **Psychedelics** |  |  |  |  |  |  |  |
| Kavain | 231.1 | 152.1 | 6.16 | 60 | 10 | 50 | 12 |
| LSD | 324.2 | 223.2 | 4.71 | 60 | 10 | 35 | 12 |
| Mescaline | 212.1 | 195.2 | 2.76 | 60 | 10 | 20 | 12 |
| **Stimulants** |  |  |  |  |  |  |  |
| 7-Hydroxymitragynine | 415.2 | 190.1 | 4.63 | 85 | 10 | 50 | 10 |
| Caffeine | 195 | 138 | 3.59 | 36 | 10 | 27 | 10 |
| Cotinine | 177 | 80 | 2.98 | 70 | 10 | 28 | 10 |
| Mitragynine | 399.2 | 174.1 | 5.43 | 71 | 10 | 59 | 12 |
| Nicotine | 163.1 | 130.1 | 1.55 | 60 | 10 | 20 | 12 |
| **Drugs** |  |  |  |  |  |  |  |
| 10-Hydroxycarbamazepine | 255.1 | 194.1 | 4.69 | 55 | 10 | 50 | 12 |
| 4-Benzamidosalicyclic acid | 258.1 | 105.1 | 4.5 | 60 | 10 | 35 | 15 |
| 9-Hydroxyrisperidone (Paliperidone) | 427.2 | 207.1 | 4.81 | 75 | 10 | 40 | 10 |
| Acebutolol | 337.2 | 116.3 | 3.88 | 56 | 10 | 27 | 10 |
| Aceclidine | 170.1 | 128.1 | 1.54 | 60 | 10 | 35 | 12 |
| Acemetacin | 416.1 | 139.1 | 7.06 | 60 | 10 | 20 | 12 |
| Acepromazine | 327 | 254 | 5.81 | 80 | 10 | 20 | 10 |
| Aceprometazine | 327.2 | 86.1 | 5.69 | 60 | 10 | 35 | 12 |
| Acetaminodantrolene | 327.1 | 186.2 | 4.84 | 80 | 10 | 35 | 10 |
| Acetaminophen | 152 | 110.1 | 2.11 | 38 | 8 | 42 | 12 |
| Acetiamin | 367.1 | 122.1 | 3.74 | 60 | 10 | 35 | 12 |
| Aconitina | 646.2 | 586.6 | 6.07 | 60 | 6 | 35 | 15 |
| Actinoquinol | 254 | 145.1 | 2.42 | 80 | 10 | 50 | 10 |
| Alizapride | 316.1 | 124.1 | 2.93 | 60 | 10 | 35 | 15 |
| Almitrine | 478.3 | 203.2 | 7.64 | 60 | 10 | 20 | 15 |
| Altretamine | 211.2 | 96.1 | 5.77 | 60 | 10 | 50 | 10 |
| Amantadine | 152.1 | 135 | 3.5 | 70 | 10 | 25 | 10 |
| Amidopyrin | 232.1 | 113 | 4.09 | 60 | 10 | 20 | 15 |
| Aminopromazine | 328.2 | 212.2 | 6.11 | 60 | 10 | 20 | 15 |
| Aminorex | 163.1 | 120.1 | 2.92 | 60 | 10 | 20 | 15 |
| Amitriptyline | 278.1 | 233.1 | 5.98 | 70 | 10 | 23 | 12 |
| Amorolfine | 318.3 | 161.2 | 6.49 | 60 | 10 | 35 | 15 |
| Amoxapine | 314.1 | 271.1 | 5.83 | 80 | 10 | 35 | 10 |
| Amrinone | 188.1 | 133.1 | 2.13 | 60 | 10 | 35 | 10 |
| Antazoline | 266.2 | 91.1 | 5.11 | 60 | 10 | 35 | 10 |
| Apraclonidin | 245 | 174.2 | 2.39 | 60 | 10 | 35 | 10 |
| Aprinidine | 323.2 | 86.1 | 6.33 | 60 | 10 | 50 | 10 |
| Aripiprazole | 448.2 | 285.1 | 6.21 | 80 | 10 | 39 | 10 |
| Atomoxetine | 256.14 | 44.2 | 5.55 | 60 | 10 | 63 | 12 |
| Atorvastatin | 559.4 | 440.4 | 6.8 | 66 | 10 | 23 | 10 |
| Atropine | 290.3 | 124.3 | 3.67 | 76 | 10 | 35 | 6 |
| Azatadine | 291.2 | 248.2 | 4.95 | 60 | 10 | 35 | 10 |
| Azelastine | 382.2 | 112.1 | 5.95 | 60 | 10 | 35 | 10 |
| Baclofen | 214.1 | 151 | 2.96 | 36 | 10 | 23 | 10 |
| Bambuterol | 368.2 | 294.1 | 4.56 | 60 | 10 | 20 | 15 |
| Bamifylline | 386.2 | 297.1 | 4.58 | 60 | 10 | 35 | 15 |
| Bamipin | 281.2 | 98.1 | 5.84 | 60 | 10 | 20 | 15 |
| Barverin | 510.3 | 174.2 | 5.05 | 80 | 10 | 35 | 4 |
| Beclamide | 198.1 | 91.1 | 4.91 | 60 | 10 | 20 | 10 |
| Beclometasone dipropionate | 521.2 | 319.2 | 7.47 | 60 | 10 | 20 | 10 |
| Bendamustine | 358.1 | 228.1 | 5.52 | 80 | 10 | 50 | 10 |
| Benodanil | 324 | 231 | 5.94 | 60 | 10 | 35 | 10 |
| Benperidole | 382.2 | 165.1 | 5.07 | 60 | 10 | 35 | 10 |
| Benproperine | 310.2 | 126.1 | 6.13 | 60 | 10 | 35 | 10 |
| Benzatropine | 308.2 | 167.2 | 5.93 | 60 | 10 | 35 | 10 |
| Benzocaine | 166.2 | 138 | 4.82 | 31 | 10 | 15 | 10 |
| Benzoctamine | 250.2 | 191.2 | 5.17 | 60 | 10 | 35 | 10 |
| Benzthiazide | 432 | 91.1 | 5.68 | 80 | 10 | 50 | 10 |
| Betaine | 118.1 | 58 | 0.71 | 80 | 10 | 35 | 10 |
| Betamethasone 17-valerate | 477.3 | 355.4 | 7.1 | 80 | 10 | 20 | 10 |
| Betamethasone7-benzoate | 497.2 | 337.1 | 7.27 | 80 | 10 | 20 | 10 |
| Bethanidine | 178.1 | 91.1 | 2.94 | 80 | 10 | 35 | 10 |
| Bezafibrate | 362.1 | 316.1 | 6.23 | 60 | 10 | 35 | 10 |
| Bornaprine | 330.2 | 171.2 | 6.06 | 60 | 10 | 35 | 10 |
| Bromperidol | 420.1 | 165.1 | 5.66 | 60 | 10 | 35 | 12 |
| Brompheniramine | 319.1 | 274.1 | 5.22 | 60 | 10 | 20 | 10 |
| Bucetin | 224.1 | 108.1 | 4.66 | 60 | 10 | 35 | 10 |
| Budipine | 294.2 | 238.2 | 5.46 | 80 | 10 | 20 | 10 |
| Bunitrolol | 249.2 | 193.2 | 4 | 60 | 10 | 20 | 10 |
| Bupivacaine | 289 | 140.3 | 4.72 | 51 | 10 | 35 | 10 |
| Bupropion | 240.3 | 184.1 | 4.52 | 60 | 10 | 16 | 10 |
| Buspirone | 386.3 | 122.1 | 5.12 | 60 | 10 | 50 | 10 |
| Cafaminol | 268.1 | 223.2 | 4.24 | 60 | 10 | 35 | 10 |
| Carbamazepine | 237.1 | 194.1 | 5.56 | 50 | 10 | 45 | 10 |
| Carbamazepine-10,11-epoxide | 253.1 | 180.1 | 4.92 | 50 | 10 | 50 | 10 |
| Carbendazim | 192.1 | 160.1 | 4.09 | 60 | 10 | 20 | 10 |
| Carbuterol | 268.2 | 134.1 | 1.86 | 60 | 10 | 35 | 10 |
| Carisoprodol | 261.1 | 176 | 5.64 | 35 | 10 | 11 | 10 |
| Carteolol | 293.2 | 237.2 | 3.14 | 60 | 10 | 20 | 10 |
| Carticain | 285.1 | 86.1 | 3.93 | 60 | 10 | 20 | 12 |
| Cerivastatin | 460.3 | 356.2 | 7.06 | 60 | 10 | 50 | 10 |
| Chlorazanil | 222.1 | 153.1 | 5.35 | 60 | 10 | 35 | 10 |
| Chlorbenzoxamine | 435.2 | 201.2 | 6.9 | 60 | 10 | 20 | 10 |
| Chlorphenethiazine | 305.1 | 72.1 | 6.07 | 60 | 10 | 35 | 10 |
| Chlorpheniramine | 275.1 | 230.1 | 5.05 | 60 | 10 | 20 | 10 |
| Chlorpromazine | 319 | 58 | 6.32 | 66 | 10 | 67 | 10 |
| Cimetidine | 253.1 | 95.1 | 2.35 | 60 | 10 | 35 | 10 |
| Cinchocaine | 344.2 | 271.2 | 5.85 | 80 | 10 | 35 | 10 |
| Cinnarizine | 369.2 | 167.1 | 6.62 | 60 | 10 | 20 | 10 |
| Ciprofloxacin | 332.1 | 231.1 | 4.01 | 60 | 10 | 50 | 12 |
| Cisapride | 466.2 | 184.1 | 5.51 | 60 | 10 | 35 | 12 |
| Citalopram | 325 | 109.1 | 5.34 | 46 | 10 | 39 | 10 |
| Clemastine | 344.2 | 215.1 | 6.44 | 60 | 10 | 20 | 12 |
| Clenbuterol | 277 | 203 | 3.99 | 65 | 10 | 21 | 10 |
| Clibucaine | 315.1 | 113.5 | 5.01 | 60 | 10 | 35 | 12 |
| Climbazole | 293.1 | 197.1 | 6.37 | 66 | 10 | 20 | 12 |
| Clobetason-butyrate | 479.3 | 343.2 | 7.45 | 80 | 10 | 19 | 10 |
| Clobutinol | 256.1 | 58 | 4.91 | 60 | 10 | 35 | 10 |
| Clomipramine | 315.1 | 86.1 | 6.36 | 26 | 10 | 23 | 10 |
| Clonidine | 230 | 74.1 | 2.88 | 56 | 9 | 101 | 10 |
| Clopamide | 346.1 | 250.1 | 4.77 | 80 | 10 | 35 | 10 |
| Clopidogrel | 322.1 | 155.2 | 7.38 | 36 | 6 | 47 | 10 |
| Corticosterone | 347.2 | 329.3 | 6.26 | 60 | 10 | 20 | 12 |
| Cortisone | 361.2 | 163.2 | 5.66 | 80 | 10 | 34 | 10 |
| Croconazole | 311.1 | 69 | 6.57 | 60 | 10 | 35 | 12 |
| Cromoglicic acid | 469.1 | 207.1 | 3.45 | 60 | 10 | 35 | 10 |
| Cyamemazine | 324.2 | 100.1 | 5.82 | 60 | 10 | 35 | 10 |
| Cyclizine | 267.2 | 167.1 | 5.41 | 60 | 10 | 20 | 10 |
| Cyclobenzaprine | 276.1 | 215.1 | 5.84 | 80 | 10 | 58 | 10 |
| Cyproheptadine | 288.2 | 191.2 | 5.87 | 60 | 10 | 35 | 10 |
| Dapiprazole | 326.2 | 189.3 | 4.6 | 60 | 10 | 20 | 10 |
| Debrisoquine | 176.1 | 134.1 | 3.54 | 60 | 10 | 20 | 12 |
| Deflazacort | 442.3 | 124.1 | 6.66 | 80 | 10 | 65 | 10 |
| Desipramine | 267.1 | 72 | 5.9 | 70 | 10 | 50 | 8 |
| Desmethylclomipramine | 301.1 | 242 | 6.34 | 60 | 10 | 35 | 10 |
| Desmethyldoxepin | 266.1 | 107 | 5.45 | 70 | 10 | 29 | 8 |
| Desoxycortone 21-(3-phenylpropionate) | 463.3 | 105.1 | 8.05 | 80 | 10 | 50 | 10 |
| Desoxycortone enantate | 443.3 | 97.1 | 8.16 | 80 | 10 | 50 | 10 |
| Dexfenfluramine | 232.1 | 159.10 | 4.69 | 60 | 10 | 35 | 10 |
| Dextromethorphan | 272.1 | 171.1 | 5.28 | 70 | 10 | 50 | 10 |
| Dextropropoxyphene | 340.2 | 58 | 5.68 | 60 | 10 | 35 | 10 |
| Diaveridine | 261.1 | 245.2 | 3.12 | 60 | 10 | 35 | 10 |
| Dibenzepin | 296.2 | 251.2 | 4.71 | 80 | 10 | 20 | 10 |
| Diclofenac | 296.2 | 215 | 6.9 | 32 | 10 | 26 | 10 |
| Dicycloverine | 310.3 | 109.1 | 6.36 | 60 | 10 | 35 | 10 |
| Dienogest | 312.2 | 135.2 | 6.04 | 60 | 10 | 50 | 10 |
| Diethazine | 299.2 | 100 | 5.88 | 60 | 10 | 20 | 10 |
| Diethylcarbamazine | 200.2 | 100.1 | 2.25 | 60 | 10 | 20 | 10 |
| Dilazep | 605.3 | 195.1 | 6.02 | 60 | 10 | 50 | 10 |
| Diltiazem | 415.2 | 178.2 | 5.73 | 46 | 6 | 33 | 8 |
| Dimetindene | 293.2 | 248.2 | 5.28 | 60 | 10 | 20 | 12 |
| Diphenhydramine | 256.2 | 167.1 | 5.21 | 60 | 10 | 20 | 10 |
| Diprophylline | 255.1 | 181.2 | 2.96 | 60 | 10 | 20 | 12 |
| Dipyridamole | 505.3 | 385.3 | 5.85 | 60 | 10 | 50 | 12 |
| Dixyrazine | 428.2 | 229.4 | 6.41 | 60 | 10 | 20 | 12 |
| Donepezil | 380.1 | 91 | 5.12 | 120 | 10 | 73 | 10 |
| Doxapram | 379.2 | 292.2 | 4.89 | 60 | 10 | 35 | 12 |
| Doxepin | 280.1 | 107 | 5.45 | 70 | 10 | 45 | 10 |
| Doxylamine | 271.2 | 182.1 | 4.42 | 60 | 10 | 50 | 12 |
| Drofenine | 318.2 | 91.1 | 6.22 | 60 | 10 | 50 | 12 |
| Ephedrine | 166.1 | 148.11 | 2.56 | 80 | 10 | 19 | 10 |
| Eprosartan | 425.2 | 135.1 | 5.78 | 60 | 10 | 50 | 12 |
| Esculin | 341.1 | 179.1 | 2.77 | 60 | 10 | 20 | 12 |
| Etodolac | 288.2 | 172.1 | 6.81 | 80 | 10 | 20 | 10 |
| Etofenamate | 370.1 | 264.1 | 7.19 | 60 | 10 | 20 | 12 |
| Etomidate | 245.1 | 95.1 | 6.31 | 60 | 10 | 35 | 12 |
| Famotidine | 338.1 | 189.1 | 2.32 | 60 | 10 | 20 | 12 |
| Fedrilate | 348.2 | 100.1 | 4.75 | 60 | 10 | 50 | 12 |
| Fendiline | 316.2 | 105.1 | 6.14 | 60 | 10 | 35 | 12 |
| Fenfluramine | 232.1 | 159 | 4.69 | 60 | 10 | 35 | 15 |
| Fenfuram | 202.1 | 109 | 5.47 | 80 | 10 | 35 | 10 |
| Fenofibrate | 361 | 233.1 | 7.62 | 80 | 10 | 20 | 10 |
| Fenpipramide | 323.2 | 238.2 | 4.78 | 60 | 10 | 35 | 12 |
| Fenpiprane | 280.2 | 167.2 | 5.54 | 60 | 10 | 35 | 12 |
| Fenproporex | 189.1 | 91.1 | 3.23 | 60 | 10 | 35 | 12 |
| Fenticonazole | 455.1 | 199.1 | 7.51 | 60 | 10 | 50 | 12 |
| Fexofenadine | 502.3 | 466.2 | 5.95 | 60 | 10 | 35 | 12 |
| Flecainide | 415.1 | 398.1 | 5.15 | 60 | 10 | 20 | 12 |
| Fluanisone | 357.2 | 165 | 5.43 | 60 | 10 | 35 | 12 |
| Fluconazole | 307.1 | 220.2 | 4.31 | 60 | 10 | 20 | 12 |
| Fludrocortisone Acetate | 423.2 | 239.2 | 6.19 | 80 | 10 | 34 | 10 |
| Fluoxetine | 310.1 | 91.1 | 5.9 | 60 | 10 | 50 | 12 |
| Fluphenazine | 438.2 | 171.2 | 6.64 | 60 | 10 | 35 | 15 |
| Fluticasone Propionate | 501.2 | 293.2 | 7.18 | 80 | 10 | 22 | 10 |
| Fluvoxamine | 319.2 | 71 | 5.77 | 60 | 10 | 20 | 15 |
| Fuberidazole | 185.1 | 157.1 | 4.64 | 60 | 10 | 35 | 15 |
| Furalaxyl | 302.1 | 95 | 6.33 | 60 | 10 | 35 | 15 |
| Galantamine | 288.2 | 213.2 | 2.4 | 60 | 10 | 35 | 15 |
| Gallopamil | 485.3 | 165.1 | 5.64 | 60 | 10 | 35 | 15 |
| Glibenclamide | 494.2 | 369 | 6.94 | 90 | 10 | 18 | 10 |
| Glimepiride | 491.2 | 352 | 7.19 | 90 | 10 | 19 | 10 |
| Glipizide | 446.2 | 321.2 | 6.17 | 85 | 10 | 20 | 10 |
| Gliquidone | 528.2 | 403.1 | 7.46 | 60 | 10 | 20 | 12 |
| Griseofulvin | 353.1 | 69 | 6.37 | 60 | 10 | 35 | 12 |
| Guaifenesin | 199.1 | 125.1 | 4.14 | 60 | 10 | 20 | 12 |
| Guanoxan | 208.1 | 100.1 | 3.71 | 60 | 10 | 35 | 12 |
| Haloperidol | 376.2 | 165.1 | 5.55 | 70 | 10 | 33 | 12 |
| Heptaminol | 146.2 | 128.1 | 1.68 | 60 | 10 | 20 | 12 |
| Hexobendine | 593.3 | 253.2 | 5.97 | 60 | 10 | 50 | 12 |
| Homatropine | 276.2 | 142.2 | 3.07 | 60 | 10 | 35 | 12 |
| Hydrocortisone | 363.2 | 121.1 | 5.66 | 80 | 10 | 31 | 10 |
| Hydroxybupropion | 256.3 | 130 | 4.39 | 54 | 10 | 61 | 12 |
| Hydroxyzine | 375.2 | 201.2 | 6.01 | 90 | 10 | 50 | 10 |
| Imipramine | 281.1 | 86 | 5.9 | 80 | 10 | 25 | 10 |
| Indanazoline | 202.1 | 115.1 | 3.93 | 60 | 10 | 50 | 15 |
| Indapamide | 366.1 | 132.2 | 5.34 | 56 | 8 | 23 | 10 |
| Indinavir | 614.4 | 421.3 | 6.3 | 60 | 10 | 50 | 15 |
| Indomethacin | 358.2 | 139 | 7.19 | 60 | 10 | 27 | 10 |
| Indoramin | 348.2 | 144.2 | 4.84 | 60 | 10 | 50 | 12 |
| Iopodic acid | 598.8 | 426.8 | 6.73 | 60 | 10 | 50 | 12 |
| Iprazochrome | 265.1 | 192.2 | 3.69 | 60 | 10 | 20 | 12 |
| Isoaminile | 245.2 | 91.1 | 4.77 | 60 | 10 | 50 | 15 |
| Isoconazole | 415 | 159 | 6.95 | 60 | 10 | 50 | 15 |
| Isoniazide | 138.1 | 121.1 | 1.23 | 80 | 10 | 20 | 10 |
| Isothipendyl | 286.1 | 241.1 | 5.4 | 60 | 10 | 20 | 12 |
| Ketoprofen | 255.2 | 209.1 | 6.26 | 66 | 10 | 20 | 10 |
| Ketorolac | 256.1 | 105.1 | 5.83 | 60 | 10 | 35 | 12 |
| Ketotifen | 310.1 | 96.1 | 4.8 | 60 | 10 | 35 | 12 |
| Lacosamide | 251.1 | 108 | 4.1 | 35 | 10 | 13 | 11 |
| Lamotrigine | 256.1 | 211.1 | 4.19 | 30 | 10 | 28 | 11 |
| Levamisole | 205 | 178 | 3.04 | 110 | 10 | 29 | 10 |
| Levetiracetam | 171.1 | 126.1 | 2.49 | 30 | 10 | 21 | 10 |
| Levomepromazine | 329.2 | 100.1 | 6.07 | 60 | 10 | 35 | 12 |
| Levopropylhexedrin | 156.2 | 69.1 | 4.41 | 60 | 10 | 20 | 12 |
| Lidocaine | 235.2 | 86.1 | 3.43 | 60 | 10 | 20 | 12 |
| Lisurid | 339.2 | 223.2 | 4.92 | 80 | 10 | 20 | 10 |
| Lonazolac | 313.1 | 267.1 | 6.94 | 60 | 10 | 35 | 10 |
| Loperamide | 477.1 | 266.2 | 6.35 | 60 | 10 | 20 | 12 |
| Loratadine | 383.1 | 337.1 | 7.46 | 60 | 10 | 35 | 15 |
| Losartan | 424.2 | 208.2 | 6.12 | 51 | 8 | 27 | 10 |
| Loxapine | 328.1 | 271.1 | 5.88 | 60 | 10 | 35 | 12 |
| Maprotiline | 278.1 | 250.1 | 5.93 | 41 | 10 | 27 | 10 |
| Mebeverine | 430.3 | 121.1 | 5.63 | 60 | 10 | 50 | 12 |
| Mecloxamine | 318.2 | 215.1 | 6.12 | 60 | 10 | 20 | 12 |
| Meclozine (meclizine) | 391.2 | 201.1 | 6.87 | 60 | 10 | 35 | 12 |
| Mefexamide | 281.2 | 208.2 | 3.7 | 60 | 10 | 20 | 12 |
| Mefloquine | 379.1 | 361.1 | 6.06 | 60 | 10 | 35 | 12 |
| Mefruside | 383 | 285 | 5.51 | 40 | 10 | 20 | 12 |
| Meloxicam | 352.1 | 115 | 6.03 | 50 | 10 | 24 | 10 |
| Memantine | 180.2 | 163.1 | 4.84 | 55 | 10 | 20 | 10 |
| Mepivacaine | 247.2 | 98.1 | 3.58 | 60 | 10 | 35 | 12 |
| Meprobamate | 219 | 158 | 4.56 | 26 | 10 | 11 | 8 |
| Meptazinol | 234.2 | 107.1 | 3.94 | 60 | 10 | 35 | 12 |
| Mequitazine | 323.1 | 212.1 | 6.23 | 60 | 10 | 35 | 12 |
| Mesoridazine | 387.2 | 98.1 | 5.63 | 60 | 10 | 50 | 12 |
| Metaxalone | 222.1 | 161.3 | 5.76 | 50 | 10 | 18 | 12 |
| Metenolone acetate | 345.2 | 187.2 | 7.62 | 60 | 10 | 20 | 12 |
| Methaphenilene | 261.1 | 97 | 5.16 | 60 | 10 | 20 | 15 |
| Methazolamide | 237 | 195.1 | 3.5 | 60 | 10 | 20 | 12 |
| Methocarbamol | 242.1 | 118.1 | 4.36 | 60 | 10 | 20 | 12 |
| Methylephedrine | 180.1 | 162.11 | 2.78 | 60 | 10 | 20 | 12 |
| Methylphenidate | 234.1 | 84.1 | 4.19 | 31 | 10 | 60 | 10 |
| Metixene | 310.2 | 197.1 | 6.19 | 60 | 10 | 35 | 12 |
| Metoclopramide | 300.1 | 227.1 | 3.81 | 60 | 10 | 25 | 12 |
| Metronidazole | 172.2 | 128 | 2.81 | 50 | 10 | 20 | 10 |
| Metyrapone | 227.1 | 120.1 | 5.03 | 60 | 10 | 50 | 15 |
| Mexiletine | 180.1 | 58 | 4.32 | 60 | 10 | 20 | 15 |
| Miconazole | 417 | 159.1 | 7.15 | 60 | 10 | 50 | 15 |
| Minoxidil | 210.1 | 164.2 | 3.85 | 60 | 10 | 35 | 10 |
| Mirtazapine | 266.2 | 195.2 | 4.54 | 60 | 10 | 35 | 12 |
| Mizolastine | 433.2 | 308.2 | 6.37 | 60 | 10 | 35 | 15 |
| Moclobemide | 269.1 | 182.1 | 3.93 | 60 | 10 | 20 | 12 |
| Mofebutazone | 233.1 | 160.2 | 4.45 | 80 | 10 | 20 | 10 |
| Molsidomine | 243.1 | 86.1 | 4.05 | 60 | 10 | 20 | 15 |
| Moperone | 356.2 | 123.1 | 5.36 | 80 | 10 | 50 | 10 |
| Moxaverine | 308.2 | 292.2 | 5.51 | 60 | 10 | 35 | 15 |
| Moxisylyte | 280.1 | 72.1 | 4.92 | 60 | 10 | 35 | 15 |
| Moxonidine | 243.2 | 207.2 | 2.34 | 66 | 8 | 19 | 10 |
| Nabumetone | 229.1 | 171.1 | 6.62 | 40 | 10 | 23 | 10 |
| Naftifine | 288.2 | 117.1 | 5.84 | 60 | 10 | 20 | 10 |
| Nalbuphine | 358.1 | 340.1 | 3.53 | 71 | 10 | 31 | 10 |
| Nalorphine | 312.2 | 201.2 | 2.75 | 60 | 10 | 35 | 12 |
| Nandrolone | 275.2 | 109.1 | 6.38 | 60 | 10 | 35 | 12 |
| Naphazoline | 211.1 | 141.1 | 4.07 | 60 | 10 | 35 | 12 |
| N-Despropylpropafenone | 300.2 | 91.1 | 5.42 | 60 | 10 | 50 | 15 |
| Nefazodone | 470.2 | 180.2 | 6.55 | 80 | 10 | 50 | 10 |
| Nicardipine | 480.2 | 315.1 | 5.86 | 60 | 10 | 35 | 12 |
| Nicotinamide | 123.1 | 80.1 | 1.52 | 60 | 10 | 35 | 12 |
| Nifedipine | 347.1 | 254.1 | 6.21 | 60 | 10 | 20 | 12 |
| Nifenazone | 309.1 | 106.1 | 4.04 | 60 | 10 | 35 | 12 |
| Nimodipine | 419.2 | 343.2 | 6.86 | 60 | 10 | 20 | 12 |
| Nimorazole | 227.1 | 114.1 | 3.33 | 60 | 10 | 20 | 12 |
| Nimustine | 273.1 | 165.2 | 3.37 | 80 | 10 | 20 | 10 |
| Nitrendipine | 361.1 | 315.1 | 6.72 | 60 | 10 | 20 | 15 |
| N-Methylephedrine | 180.1 | 162.1 | 2.77 | 60 | 10 | 20 | 15 |
| Nomifensine | 239.2 | 196.2 | 4.55 | 60 | 10 | 20 | 15 |
| Norethisterone acetate | 341.2 | 281.3 | 7.16 | 60 | 10 | 20 | 12 |
| Nortriptyline | 264.1 | 233.1 | 5.98 | 56 | 10 | 17 | 10 |
| Noscapine | 414.2 | 220.2 | 5.26 | 60 | 10 | 35 | 12 |
| Olanzapine | 313.1 | 256.1 | 4 | 70 | 10 | 33 | 10 |
| Ondansetron | 294.2 | 170.2 | 4.66 | 60 | 10 | 35 | 12 |
| Ornidazole | 220 | 128 | 4.24 | 70 | 10 | 23 | 10 |
| Orphenadrine | 270.2 | 181.2 | 5.55 | 60 | 10 | 20 | 15 |
| Oxatomide | 427.2 | 167.3 | 6.18 | 60 | 10 | 20 | 12 |
| Oxeladin | 336.3 | 100.1 | 5.82 | 60 | 10 | 35 | 15 |
| Oxilofrine | 182.1 | 164.1 | 1.19 | 60 | 10 | 20 | 12 |
| Oxomemazine | 331.1 | 58 | 4.78 | 60 | 10 | 50 | 12 |
| Oxybuprocaine | 309.2 | 192.2 | 5.04 | 60 | 10 | 20 | 12 |
| Oxybutynin | 358.2 | 142.2 | 5.98 | 60 | 10 | 35 | 12 |
| Oxyfedrine | 314.2 | 105.1 | 5.11 | 60 | 10 | 35 | 10 |
| Oxymetazoline | 261.2 | 205.2 | 5.17 | 60 | 10 | 35 | 12 |
| Oxypendyl | 371.2 | 241.1 | 5.81 | 40 | 10 | 35 | 12 |
| Oxypertine | 380.2 | 175.2 | 5.03 | 60 | 10 | 20 | 12 |
| Paraoxon | 276.1 | 220.1 | 6.03 | 60 | 10 | 20 | 15 |
| Penfluridol | 524.2 | 109 | 6.71 | 60 | 10 | 50 | 15 |
| Pentamidine | 341.2 | 120.1 | 4.01 | 60 | 10 | 50 | 15 |
| Pentazocine | 286.3 | 175.1 | 4.55 | 30 | 10 | 35 | 8 |
| Pentoxifylline | 279.1 | 181.2 | 4.79 | 68 | 10 | 20 | 12 |
| Pentoxyverine | 334.2 | 100.1 | 5.79 | 60 | 10 | 35 | 12 |
| Perazine | 340.2 | 141.2 | 6.27 | 60 | 10 | 20 | 12 |
| Pergolide | 315.2 | 208.2 | 5.47 | 40 | 10 | 35 | 12 |
| Periciazine | 366.1 | 142.2 | 5.81 | 60 | 10 | 35 | 12 |
| Perphenazine | 404.1 | 171.2 | 6.61 | 60 | 10 | 35 | 12 |
| Phenazopyridine | 214.1 | 122.1 | 5.57 | 60 | 10 | 20 | 12 |
| Pheniramine | 241.2 | 196.21 | 4.24 | 60 | 10 | 20 | 10 |
| Phenprobamate | 180.1 | 91 | 5.45 | 60 | 10 | 20 | 10 |
| Phentermine | 149.9 | 90.9 | 3.38 | 40 | 10 | 25 | 10 |
| Phenylpropanolamine (norefedrina) | 152.1 | 134.1 | 2.18 | 60 | 10 | 20 | 10 |
| Phenyltoloxamine | 256.2 | 72 | 5.54 | 60 | 10 | 35 | 12 |
| Phenytoin | 253.1 | 225.1 | 5.46 | 65 | 10 | 16 | 11 |
| Physostigmine | 276.2 | 162.2 | 3.28 | 60 | 10 | 20 | 15 |
| Pilocarpine | 209.1 | 95.1 | 2.12 | 60 | 10 | 50 | 15 |
| Pioglitazone | 357.1 | 134.2 | 6.34 | 60 | 10 | 35 | 15 |
| Pipamperone | 376.2 | 165.2 | 4.97 | 60 | 10 | 35 | 15 |
| Piprozolin | 299.1 | 214.1 | 6.8 | 60 | 10 | 20 | 10 |
| Piracetam | 143.1 | 98.1 | 1.16 | 60 | 10 | 20 | 10 |
| Prenylamine | 330.2 | 91.1 | 6.29 | 60 | 10 | 50 | 10 |
| Prilocaine | 221.2 | 86.1 | 3.56 | 60 | 10 | 20 | 15 |
| Procainamide | 236.2 | 163.2 | 2.1 | 60 | 10 | 20 | 12 |
| Procaine | 237.2 | 100.1 | 2.7 | 60 | 10 | 20 | 10 |
| Prochlorperazine | 374.1 | 141.1 | 6.74 | 60 | 10 | 35 | 12 |
| Procyclidine | 288.2 | 270.2 | 5.61 | 50 | 10 | 21 | 10 |
| Progesterone | 315.2 | 109.1 | 7.25 | 60 | 10 | 35 | 12 |
| Promazine | 285.1 | 86.11 | 5.85 | 60 | 10 | 20 | 15 |
| Propafenone | 342.2 | 116.1 | 5.88 | 60 | 10 | 35 | 12 |
| Propiconazole | 342.1 | 159 | 7.14 | 60 | 10 | 50 | 12 |
| Propionylpromazine | 341 | 268 | 6.19 | 77 | 10 | 20 | 10 |
| Propipocaine | 276.2 | 98.1 | 5.19 | 60 | 10 | 35 | 12 |
| Propiverine | 368.2 | 105.1 | 6.35 | 60 | 10 | 50 | 12 |
| Protriptyline | 264.1 | 155 | 5.83 | 76 | 10 | 29 | 10 |
| Pseudoephedrine | 166.1 | 148.1 | 2.61 | 80 | 10 | 19 | 10 |
| Pyribenzamine | 256.2 | 211.2 | 4.98 | 60 | 10 | 20 | 12 |
| Pyridoxine | 170.1 | 134.1 | 1.21 | 60 | 10 | 20 | 12 |
| Pyrilamine | 286.2 | 121.1 | 5.04 | 70 | 10 | 35 | 10 |
| Pyrimethamine | 249.1 | 233.1 | 4.76 | 60 | 10 | 35 | 12 |
| Quinidine | 285 | 205.1 | 1.63 | 66 | 10 | 25 | 10 |
| Quinine | 325.2 | 79.1 | 4.71 | 60 | 10 | 50 | 12 |
| Ramifenazone | 246.2 | 125.1 | 4.12 | 60 | 10 | 20 | 10 |
| Raubasine | 353.2 | 144.1 | 5.14 | 80 | 10 | 35 | 10 |
| Reproterol | 390.2 | 221.2 | 2.98 | 60 | 10 | 35 | 15 |
| Riluzole | 235 | 166.1 | 5.96 | 60 | 10 | 35 | 15 |
| Risperidone | 411.2 | 191.1 | 5.13 | 75 | 10 | 40 | 10 |
| Rizatriptan | 270.2 | 201.2 | 2.84 | 60 | 10 | 20 | 15 |
| Ropinirole | 261.2 | 114.2 | 3.39 | 60 | 10 | 35 | 15 |
| Ropivacaine | 275.2 | 126.1 | 4.25 | 60 | 10 | 35 | 15 |
| Rosiglitazon | 358.1 | 135.1 | 5.1 | 60 | 10 | 35 | 15 |
| Salicylamide | 138.1 | 121 | 3.59 | 60 | 10 | 20 | 15 |
| Scopolamine | 304.3 | 138.2 | 3.1 | 61 | 10 | 29 | 8 |
| Sertindole | 441.2 | 113 | 6.54 | 60 | 10 | 50 | 12 |
| Sertraline | 306.1 | 159 | 6.26 | 60 | 10 | 35 | 12 |
| Sibutramine | 280.1 | 125.1 | 5.87 | 40 | 10 | 35 | 12 |
| Sildenafil | 475.2 | 58 | 5.79 | 60 | 10 | 50 | 15 |
| Stanozolol | 329.3 | 81 | 6.9 | 60 | 10 | 50 | 12 |
| Sulfabenzamide | 277.1 | 156 | 4.23 | 60 | 10 | 19 | 10 |
| Sulfacetamide | 215 | 156 | 2.23 | 52 | 10 | 17 | 10 |
| Sulfaclomide | 313.1 | 158.1 | 3.48 | 80 | 10 | 35 | 10 |
| Sulfadiazine | 251.1 | 156 | 2.82 | 40 | 10 | 22 | 10 |
| Sulfadicramide | 255.1 | 92 | 3.94 | 80 | 10 | 35 | 10 |
| Sulfadoxine | 311.1 | 156.1 | 4.19 | 70 | 10 | 30 | 10 |
| Sulfaethidole | 285 | 156.1 | 4.37 | 60 | 10 | 20 | 12 |
| Sulfamerazine | 265.2 | 156.1 | 3.3 | 82 | 10 | 25 | 10 |
| Sulfamethizole | 271 | 156.1 | 3.67 | 65 | 10 | 21 | 10 |
| Sulfamethoxazole | 254.1 | 156 | 4.01 | 65 | 10 | 22 | 10 |
| Sulfapyridine | 250.1 | 156.1 | 3.12 | 40 | 10 | 23 | 10 |
| Sulfasalazine | 399.1 | 119 | 5.37 | 80 | 10 | 50 | 10 |
| Sulfathiazole | 256 | 156 | 3.03 | 40 | 10 | 22 | 10 |
| Suxibuzone | 439.2 | 309.2 | 6.81 | 60 | 10 | 20 | 15 |
| Tacrine | 199.1 | 144.2 | 3.89 | 80 | 10 | 50 | 10 |
| Tadalafil | 390.1 | 268.1 | 6.51 | 60 | 10 | 20 | 15 |
| Tamoxifen | 372.2 | 72.1 | 6.93 | 60 | 10 | 50 | 15 |
| Telmisartan | 515.2 | 276.3 | 7.12 | 96 | 8 | 65 | 10 |
| Terazosine | 388.1 | 290.3 | 4.24 | 76 | 10 | 29 | 10 |
| Terbutaline | 226.1 | 152 | 2.03 | 60 | 10 | 20 | 15 |
| Terconazole | 532.2 | 219.1 | 6.31 | 60 | 10 | 20 | 15 |
| Terfenadine | 472.2 | 436.2 | 6.7 | 60 | 10 | 35 | 12 |
| Testosterone benzoate | 393.2 | 105.1 | 8.08 | 88 | 10 | 50 | 10 |
| Tetracaine | 265.2 | 176.2 | 5.06 | 60 | 10 | 20 | 15 |
| Tetroxoprim | 335.2 | 230.2 | 3.53 | 60 | 10 | 35 | 12 |
| Theophylline | 181.1 | 96 | 2.91 | 50 | 10 | 30 | 11 |
| Thiethylperazine | 400.2 | 141.2 | 7 | 90 | 10 | 35 | 10 |
| Thioproperazine | 447.2 | 141.2 | 6.25 | 60 | 10 | 35 | 12 |
| Thioridazine | 371.2 | 98.1 | 6.7 | 60 | 10 | 50 | 12 |
| Thiothixene | 444.2 | 98.1 | 6.4 | 80 | 10 | 50 | 10 |
| Tiagabine | 376.1 | 111 | 6.16 | 30 | 10 | 55 | 11 |
| Ticlopidine | 264.1 | 125 | 5.16 | 60 | 10 | 50 | 15 |
| Timolol | 317.1 | 261.2 | 4.2 | 60 | 10 | 20 | 15 |
| Tinidazole | 248.2 | 121.2 | 3.65 | 80 | 10 | 21 | 10 |
| Tolazamide | 312.1 | 115.1 | 6.07 | 80 | 10 | 20 | 10 |
| Tolazoline | 161.1 | 91 | 2.44 | 60 | 10 | 50 | 15 |
| Tolbutamide | 271.1 | 155 | 5.75 | 72 | 10 | 24 | 10 |
| Toliprolol | 224.2 | 91 | 4.34 | 60 | 10 | 50 | 15 |
| Tolmetin | 258.2 | 119.1 | 6.03 | 60 | 10 | 24 | 10 |
| Tolnaftate | 308.1 | 148.1 | 7.59 | 60 | 10 | 20 | 15 |
| Tolpropamine | 254.2 | 165.2 | 5.59 | 60 | 10 | 50 | 15 |
| Tolycaine | 279.2 | 86.1 | 3.64 | 60 | 10 | 35 | 15 |
| Topiramate | 357.2 | 282 | 4.84 | 30 | 10 | 18 | 11 |
| Torasemide | 349.1 | 264.1 | 5.32 | 60 | 10 | 20 | 15 |
| Tranexamic acid | 158.1 | 95.1 | 0.83 | 60 | 10 | 20 | 15 |
| Tranylcypromine | 134.1 | 117 | 5.24 | 80 | 10 | 20 | 10 |
| Trapidil | 206.1 | 109 | 5.17 | 80 | 10 | 50 | 10 |
| Trazodone | 372.2 | 176.1 | 5.31 | 60 | 10 | 35 | 15 |
| Triamterene | 254.2 | 237.2 | 3.95 | 76 | 12 | 33 | 10 |
| Trifluoperazine | 408 | 141.2 | 6.74 | 60 | 10 | 35 | 12 |
| Triflupromazine | 353.1 | 86.1 | 6.38 | 60 | 10 | 35 | 12 |
| Trihexyphenidyl | 302.2 | 99.4 | 5.75 | 80 | 10 | 35 | 10 |
| Trimethobenzamide | 389.2 | 72.1 | 4.16 | 60 | 10 | 35 | 15 |
| Trimipramine | 295.2 | 100.1 | 6.06 | 60 | 10 | 35 | 12 |
| Tripelennamine | 256.2 | 211.1 | 4.97 | 60 | 10 | 20 | 12 |
| Triprolidine | 279.2 | 208.1 | 5.27 | 60 | 10 | 20 | 12 |
| Tritoqualine | 501.2 | 220.1 | 6.89 | 60 | 10 | 35 | 12 |
| Tromantadine | 281.2 | 135.1 | 5.02 | 60 | 10 | 35 | 12 |
| Tropisetron | 285.2 | 124.1 | 4.41 | 60 | 10 | 35 | 12 |
| Tulobuterol | 228 | 154 | 4.22 | 65 | 10 | 21 | 10 |
| Valdecoxib | 315.1 | 132.1 | 5.83 | 60 | 10 | 35 | 15 |
| Valsartan | 436.2 | 207.1 | 6.44 | 60 | 10 | 35 | 12 |
| Vardenafil | 489.2 | 151.2 | 5.83 | 60 | 10 | 50 | 12 |
| Venlafaxine | 278.2 | 58 | 4.72 | 60 | 10 | 35 | 12 |
| Verapamil | 455.3 | 165.1 | 5.6 | 60 | 10 | 35 | 12 |
| Vincamine | 355.2 | 337.3 | 4.8 | 60 | 10 | 20 | 15 |
| Voriconazole | 350.3 | 224.3 | 6 | 80 | 10 | 23 | 12 |
| Warfarin | 309.1 | 163.1 | 6.48 | 71 | 8 | 19 | 10 |
| Xipamide | 355.1 | 122.2 | 5.72 | 60 | 10 | 35 | 15 |
| Xylometazoline | 245.2 | 145.1 | 5.53 | 60 | 10 | 50 | 15 |
| Yohimbine | 355.2 | 144.1 | 4.5 | 60 | 10 | 35 | 15 |
| Ziprasidone | 413 | 194 | 5.56 | 80 | 10 | 37 | 10 |
| Zonisamide | 213.1 | 51 | 3.8 | 55 | 10 | 80 | 10 |
| Zotepine | 332.1 | 72.1 | 6.37 | 89 | 10 | 35 | 10 |
| **Amino acids** |  |  |  |  |  |  |  |
| 5-Hydroxytryptophan | 221.1 | 162 | 1.39 | 30 | 10 | 16 | 12 |
| Adenosine | 268.1 | 136.1 | 1.99 | 80 | 10 | 35 | 10 |
| **Pesticides** |  |  |  |  |  |  |  |
| 2-Benzyltetronic acid | 191.1 | 113 | 4.23 | 60 | 10 | 35 | 12 |
| Alachlor | 270.1 | 238.2 | 6.79 | 60 | 10 | 20 | 15 |
| Ametryn | 228.1 | 186.1 | 6.11 | 80 | 10 | 20 | 10 |
| Atraton | 212.2 | 170.2 | 5.36 | 60 | 10 | 20 | 10 |
| Benzthiazuron | 208.1 | 151.1 | 5.25 | 60 | 10 | 20 | 10 |
| Brodifacoum | 525 | 337 | 8.12 | 60 | 10 | 50 | 10 |
| Bromacil | 261 | 205 | 5.37 | 60 | 10 | 20 | 10 |
| Buprofezin | 306.2 | 106.1 | 7.45 | 60 | 10 | 50 | 10 |
| Carbaril | 215.1 | 197.2 | 5.48 | 80 | 10 | 20 | 10 |
| Coumatetralyl | 293.1 | 175.1 | 6.62 | 60 | 10 | 35 | 12 |
| Demeton-O-methyl | 231 | 155.1 | 0.85 | 80 | 10 | 20 | 10 |
| Demeton-S-methylsulfone | 263 | 169.1 | 3.48 | 60 | 10 | 20 | 10 |
| Desmedipham | 301.1 | 136.11 | 6.06 | 60 | 10 | 35 | 10 |
| Difenoxuron | 287.1 | 72.1 | 6.08 | 60 | 10 | 35 | 10 |
| Dimefuron | 339.1 | 72.1 | 6.1 | 80 | 10 | 50 | 12 |
| Dimethachlor | 256.1 | 224.2 | 6.18 | 80 | 10 | 20 | 12 |
| Diphenamid | 240.1 | 134.1 | 6.22 | 60 | 10 | 35 | 12 |
| Flurochloridone | 312 | 292 | 6.83 | 90 | 10 | 29 | 12 |
| Haloxyfop ethoxyethyl ester | 434.1 | 316 | 7.49 | 60 | 10 | 20 | 12 |
| Hexazinone | 253.2 | 171.2 | 5.6 | 60 | 10 | 20 | 12 |
| Isoproturon | 207.1 | 72 | 5.76 | 60 | 10 | 35 | 12 |
| Metamitron | 203.1 | 175.2 | 4.39 | 60 | 10 | 20 | 12 |
| Metazachlor | 278.1 | 134.1 | 6.07 | 60 | 10 | 20 | 12 |
| Methabenzthiazuron | 222.1 | 165.1 | 5.87 | 60 | 10 | 20 | 12 |
| Methoprotryne | 272.2 | 240.2 | 6.18 | 60 | 10 | 20 | 12 |
| Monolinuron | 215.1 | 126 | 5.59 | 60 | 10 | 20 | 15 |
| Monuron | 199.1 | 126 | 5.11 | 40 | 10 | 35 | 12 |
| N,N-Diethyl-m-toluamide | 192.1 | 119 | 5.83 | 80 | 10 | 20 | 10 |
| Napropamide | 272.2 | 171.2 | 6.78 | 80 | 10 | 20 | 10 |
| Oxadixyl | 279.1 | 219.2 | 5.31 | 60 | 10 | 20 | 15 |
| Phenmedipham | 301.1 | 136.1 | 6.1 | 60 | 10 | 20 | 10 |
| Prometryn | 242.1 | 200.2 | 6.46 | 60 | 10 | 20 | 12 |
| Strychnine | 335 | 184.1 | 4.11 | 80 | 10 | 49 | 10 |
| Terbutryn | 242.1 | 186.2 | 6.52 | 60 | 10 | 20 | 15 |
| Thiabendazole | 202.2 | 175 | 4.73 | 60 | 10 | 37 | 10 |
| Triallate | 304 | 91 | 7.53 | 60 | 10 | 50 | 15 |
| **Synthetic cannabinoids** |  |  |  |  |  |  |  |
| 4-fluoro-MDMB-BUTICA | 363.2 | 218.1 | 6.96 | 57 | 10 | 22 | 12 |
| 5-Chloro AB-PINACA | 366 | 249 | 6.84 | 50 | 10 | 30 | 12 |
| 5-chloro THJ 018 | 377.2 | 248.9 | 7.87 | 60 | 10 | 24 | 12 |
| 5F-APINACA (5F-AKB48) | 384.3 | 135 | 7.83 | 60 | 10 | 22 | 12 |
| 5F-APP-PICA | 396.3 | 232 | 6.75 | 60 | 10 | 45 | 12 |
| 5F-APP-PINACA | 397.3 | 233 | 6.78 | 60 | 10 | 45 | 12 |
| 5F-Cumyl-PINACA | 368.3 | 250 | 7.4 | 90 | 10 | 11 | 12 |
| 5-fluoro CUMYL-PICA | 367.3 | 249.1 | 7.17 | 73 | 10 | 22 | 12 |
| 5-fluoro EDMB-PICA | 391.2 | 232.1 | 7.29 | 71 | 9 | 24 | 12 |
| 5-Fluoro EMB-PICA | 377.3 | 232.1 | 7.08 | 60 | 10 | 20 | 12 |
| 5-fluoro MDMB-PICA | 377.1 | 232.1 | 7.14 | 58 | 10 | 21 | 12 |
| 5-fluoro NNEI 2-naphthyl isomer | 375.3 | 232 | 7.29 | 40 | 10 | 20 | 12 |
| 5-fluoro CUMYL-P7AICA | 368.1 | 250.2 | 6.82 | 72 | 10 | 23 | 12 |
| 5-fluoro CUMYL-PeGACLONE | 391.1 | 273.2 | 7.37 | 48 | 10 | 18 | 12 |
| AB-CHMINACA | 357.4 | 241.2 | 7.11 | 80 | 10 | 34 | 12 |
| AB-FUBINACA | 369.3 | 253 | 6.64 | 60 | 10 | 33 | 12 |
| ADB-4en-PINACA | 343.3 | 298.2 | 6.83 | 53 | 7 | 20 | 12 |
| ADB-Fubinaca | 383.4 | 253 | 6.85 | 90 | 10 | 40 | 12 |
| AM 2201 (N-4-hydroxypentyl) | 376.1 | 155.1 | 6.97 | 65 | 10 | 31 | 10 |
| APP-FUBINACA | 417.3 | 109 | 6.91 | 70 | 10 | 40 | 12 |
| CUMYL-PeGACLONE | 373.3 | 255 | 7.61 | 80 | 10 | 15 | 12 |
| JWH 018 N-5-pentanoic acid | 372.1 | 155.1 | 7.08 | 90 | 10 | 33 | 12 |
| JWH-007 | 356.2 | 155.1 | 7.88 | 99 | 10 | 35 | 11 |
| JWH-015 | 328 | 155 | 7.51 | 88 | 10 | 33 | 10 |
| JWH-018 4-OH pentyl | 358.1 | 155.1 | 7.03 | 100 | 10 | 37 | 12 |
| JWH-019 6-OH hexyl | 372.2 | 155.1 | 7.24 | 90 | 10 | 27 | 12 |
| JWH-073 3-OH butyl | 344.1 | 155.1 | 6.94 | 100 | 10 | 33 | 12 |
| JWH-073 butanoic acid | 358.2 | 155.1 | 6.94 | 90 | 10 | 33 | 10 |
| JWH-122 | 356 | 169 | 8.02 | 89 | 11 | 35 | 12 |
| JWH-122 5-OH pentyl | 372.1 | 169.1 | 7.24 | 90 | 10 | 32 | 12 |
| JWH-200 | 385 | 155 | 7.04 | 75 | 10 | 30 | 11 |
| JWH-210 5-OH pentyl | 386.1 | 183.1 | 7.43 | 100 | 10 | 45 | 12 |
| JWH-250 | 336 | 121.01 | 7.59 | 76 | 10 | 29 | 11 |
| JWH-250 4-OH pentyl | 352.1 | 121.1 | 6.69 | 90 | 10 | 35 | 12 |
| MDMB-4en-PICA | 357.2 | 212.1 | 7.23 | 52 | 10 | 20 | 12 |
| MDMB-4en-PINACA | 358.2 | 213.2 | 7.37 | 65 | 10 | 34 | 12 |
| MDMB-4en-PINACA Butanoic COOH | 344.2 | 213.2 | 6.96 | 65 | 10 | 34 | 12 |
| MDMB-Chimica | 385.5 | 240 | 7.59 | 80 | 10 | 26 | 12 |
| MMB-2201 | 363.3 | 231.9 | 6.91 | 40 | 10 | 20 | 12 |
| UR-144 | 312.1 | 125.2 | 7.65 | 82 | 10 | 31 | 12 |
| **Synthetic opioids** |  |  |  |  |  |  |  |
| 2-methyl AP-237 | 287.1 | 117.1 | 5.38 | 60 | 10 | 23 | 12 |
| Acetyl Fentanyl | 323.1 | 188.1 | 4.72 | 90 | 10 | 31 | 12 |
| Alfentanil | 417.3 | 268.1 | 5.44 | 76 | 10 | 23 | 18 |
| AP-237 | 273.1 | 117 | 4.97 | 53 | 10 | 19 | 12 |
| Brorphine | 402.1 | 218.2 | 5.47 | 73 | 10 | 29 | 12 |
| Butyryl fentanyl | 351.2 | 188.1 | 5.53 | 50 | 10 | 32 | 12 |
| Butyryl Norfentanyl | 247.1 | 84.1 | 4.52 | 60 | 10 | 28 | 12 |
| Carfentanyl | 395.2 | 335 | 5.4 | 50 | 10 | 32 | 12 |
| Cyclopropyl fentanyl | 349.2 | 105 | 5.45 | 60 | 10 | 40 | 12 |
| Fentanyl | 337.1 | 188.1 | 5.15 | 90 | 10 | 31 | 10 |
| Furanyl fentanyl | 375.1 | 188.2 | 5.32 | 92 | 10 | 31 | 12 |
| Furanyl Norfentanyl | 271 | 84.1 | 4.15 | 60 | 10 | 24 | 12 |
| Isobutyryl fentanyl | 351.1 | 188.2 | 5.48 | 81 | 10 | 34 | 12 |
| Isotonitazene | 411.2 | 100.2 | 5.97 | 75 | 10 | 32 | 12 |
| Methoxyacetyl fentanyl | 353.1 | 188.2 | 4.75 | 81 | 10 | 31 | 12 |
| Metoxy acetil norfentanyl | 249 | 84 | 3.07 | 40 | 10 | 15 | 12 |
| Norfentanyl | 233.2 | 84.1 | 3.85 | 50 | 10 | 23 | 10 |
| Ocfentanyl | 371.2 | 188.2 | 4.84 | 90 | 10 | 32 | 12 |
| Para-fluoro-Furanyl fentanyl | 393.1 | 188.2 | 5.39 | 78 | 10 | 32 | 12 |
| Propoxyphene | 340.1 | 266.1 | 5.69 | 46 | 10 | 20 | 10 |
| Sufentanil | 387.1 | 238 | 5.64 | 46 | 10 | 27 | 12 |
| **Synthetic cathinones** |  |  |  |  |  |  |  |
| Trans-3-metil-Norfentanyl | 247 | 98 | 4.24 | 50 | 10 | 20 | 12 |
| 3,4-DMMC | 192.1 | 174 | 3.74 | 55 | 10 | 19 | 12 |
| 3,4-MD-alpha-PHP | 290 | 135.1 | 4.99 | 70 | 10 | 35 | 12 |
| 3-Methylmethcathinone | 178.2 | 145.1 | 3.61 | 50 | 10 | 28 | 12 |
| 4-MEC | 192.1 | 174.2 | 3.74 | 80 | 10 | 20 | 10 |
| 5-MMPA | 170.1 | 111 | 3.72 | 45 | 10 | 31 | 12 |
| Alpha-PBP (α-Pyrrolidinobutiophenone) | 218.2 | 91.1 | 3.62 | 41 | 10 | 32 | 10 |
| Alpha-PPP (α-Pyrrolidinopropiophenone) | 204.1 | 105 | 3.23 | 80 | 10 | 30 | 10 |
| Alpha-PVP | 232.1 | 91 | 4.17 | 80 | 10 | 30 | 10 |
| Alpha-Pyrrolidinohexanophenone | 246.1 | 140.2 | 4.78 | 113 | 10 | 36 | 12 |
| Buphedrone | 178.1 | 131.1 | 3.2 | 50 | 10 | 28 | 10 |
| Butylone | 222.1 | 174.1 | 3.5 | 80 | 10 | 26 | 10 |
| Cathinone | 150.1 | 117.1 | 2.36 | 80 | 10 | 33 | 10 |
| Ethcathinone | 178.1 | 160.1 | 2.91 | 80 | 10 | 18 | 10 |
| Ethylone | 222.1 | 174.12 | 3.39 | 52 | 10 | 25 | 12 |
| Eutylone | 236.1 | 188.1 | 3.89 | 54 | 10 | 26 | 12 |
| Flephedrone | 182.1 | 164.2 | 2.84 | 80 | 10 | 19 | 10 |
| MDPV | 276.1 | 175.1 | 4.43 | 70 | 10 | 30 | 10 |
| Mephedrone | 178.1 | 145.1 | 3.48 | 46 | 10 | 26 | 8 |
| Methcathinone | 164.1 | 146.1 | 2.6 | 80 | 10 | 19 | 10 |
| Methedrone | 194.1 | 161.1 | 3.23 | 50 | 10 | 26 | 10 |
| Methylone | 208.1 | 160.1 | 2.95 | 60 | 10 | 25 | 12 |
| Methylone-IS | 211.1 | 163.1 | 2.95 | 50 | 10 | 25 | 10 |
| Naphyrone | 282.2 | 141.1 | 5.47 | 80 | 10 | 37 | 10 |
| N-Ethyl pentylone | 250.1 | 202.1 | 4.42 | 62 | 10 | 26 | 12 |
| Pentedrone | 192.2 | 174.1 | 3.74 | 41 | 10 | 17 | 12 |
| Pentylone | 236.2 | 188.2 | 4.11 | 80 | 10 | 23 | 10 |
| Pyrovalerone | 246.2 | 105.1 | 4.75 | 80 | 10 | 32 | 10 |
| **Phenethylamines** |  |  |  |  |  |  |  |
| 25B-NBOMe | 381.9 | 121 | 5.9 | 31 | 10 | 33 | 10 |
| 25C-NBOMe | 336 | 121 | 5.76 | 41 | 10 | 25 | 10 |
| 25H-NBOMe | 301.9 | 121.1 | 5.28 | 21 | 10 | 27 | 10 |
| 25I-NBOMe | 428 | 121 | 6.1 | 56 | 10 | 31 | 10 |
| 2C-B | 260.1 | 243 | 4.54 | 80 | 10 | 15 | 10 |
| 2C-B-Fly | 285.1 | 189.1 | 4.81 | 51 | 10 | 35 | 10 |
| 2C-C | 216.1 | 199.1 | 4.31 | 50 | 10 | 17 | 10 |
| 2C-E | 210.2 | 193.2 | 4.85 | 80 | 10 | 16 | 10 |
| 2C-H | 182 | 165.1 | 3.35 | 80 | 10 | 15 | 10 |
| 2C-I | 308.1 | 291 | 4.89 | 80 | 10 | 18 | 10 |
| 2C-N | 227.2 | 210 | 3.65 | 60 | 10 | 17 | 10 |
| 2C-P | 224.1 | 207.1 | 5.36 | 80 | 10 | 17 | 10 |
| 2C-T-2 | 242.1 | 225.1 | 4.71 | 80 | 10 | 17 | 10 |
| 2C-T-7 | 256.1 | 197.1 | 5.25 | 80 | 10 | 20 | 10 |
| 5-APB | 176.2 | 91 | 3.95 | 20 | 10 | 38 | 12 |
| 5-APB/6-APB | 176 | 131 | 3.83 | 60 | 10 | 27 | 10 |
| 5-IAI | 260 | 116 | 4.58 | 80 | 10 | 38 | 10 |
| 6-APB | 176.21 | 91 | 3.96 | 20 | 10 | 26 | 12 |
| 6-MAPB | 190.21 | 159 | 4.09 | 40 | 10 | 10 | 12 |
| BDB  (1,3-Benzodioxolylbutanamine) | 194.1 | 147.1 | 3.37 | 30 | 10 | 18 | 12 |
| Bromo-Dragon FLY | 294 | 277 | 5.49 | 15 | 10 | 20 | 12 |
| MBDB | 208.1 | 135.2 | 3.86 | 51 | 10 | 25 | 10 |
| MDAI (5,6-Methylenedioxy-2-aminoindane) | 178.1 | 161.1 | 2.83 | 80 | 10 | 18 | 10 |
| 2-fluoro Deschloroketamine | 222.1 | 109 | 3.59 | 53 | 10 | 39 | 12 |
| 3-methoxy-PCE | 234.1 | 189.1 | 5.06 | 42 | 10 | 16 | 12 |
| Deschloro-N-ethyl-Ketamine | 218 | 173.1 | 3.91 | 54 | 10 | 19 | 12 |
| Ketamine | 238.1 | 125.1 | 3.83 | 80 | 10 | 40 | 10 |
| Ketamine-IS | 242.1 | 129.1 | 3.83 | 40 | 10 | 33 | 10 |
| Methoxetamine | 248.1 | 203.1 | 4.21 | 55 | 8 | 21 | 10 |
| Methoxpropamine | 262.2 | 203.1 | 4.65 | 63 | 10 | 21 | 12 |
| Norketamine | 224.1 | 125.1 | 3.77 | 54 | 10 | 36 | 10 |
| Phencyclidine | 244.1 | 159.1 | 4.94 | 56 | 10 | 19 | 12 |
| **Tryptamines** |  |  |  |  |  |  |  |
| 5-MeO-DALT  (N,N-Diallyl-5-methoxytryptamine ) | 271.2 | 110.1 | 4.31 | 80 | 10 | 19 | 10 |
| 5-MeO-DIPT  (5-Methoxy-N,N-diisopropyltryptamine) | 275.2 | 174.1 | 4.33 | 60 | 10 | 35 | 12 |

| 5-MeO-DMT  (5-metossi-N,N-dimetiltriptamina) | 219.1 | 58 | 3.25 | 80 | 10 | 21 | 10 |
| --- | --- | --- | --- | --- | --- | --- | --- |
| Bufotenine | 205 | 160 | 1.96 | 60 | 10 | 35 | 12 |
| Bulbocapnin | 326.1 | 295.1 | 4.07 | 80 | 10 | 20 | 10 |
| DMT (dimetiltriptamina) | 189.1 | 58 | 3.22 | 80 | 10 | 27 | 10 |
| DOB  (2,5-dimetossi-4-bromoamfetamina) | 274.1 | 257.1 | 4.76 | 80 | 10 | 20 | 10 |
| Melatonin | 233.1 | 174.1 | 4.45 | 60 | 10 | 20 | 12 |
| Psilocin | 205.1 | 58 | 1.96 | 60 | 10 | 20 | 12 |
| Psilocybin | 237.1 | 120 | 2.7 | 70 | 10 | 33 | 10 |
| **Benzimidazoles** |  |  |  |  |  |  |  |
| Butonitazene | 425.3 | 100.1 | 6.44 | 88 | 10 | 35 | 12 |
| Etodesnitazene (cytrate) | 352.3 | 100.1 | 5.34 | 70 | 10 | 27 | 12 |
| Flunitazene | 371.2 | 100.2 | 5.4 | 77 | 10 | 35 | 12 |
| Metodesnitazene | 338.3 | 100 | 4.96 | 77 | 10 | 25 | 12 |
| Metonitazene | 383.2 | 100.1 | 5.37 | 65 | 9 | 30 | 12 |
| N-Pyrrolidino Etonitazene | 395.2 | 98.1 | 5.61 | 80 | 12 | 35 | 12 |
| **Piperazines** |  |  |  |  |  |  |  |
| Benzylpiperazine BZP | 177.2 | 91.1 | 3.19 | 80 | 10 | 32 | 10 |
| TFMPP | 231.1 | 188.1 | 4.74 | 80 | 10 | 30 | 10 |

**Tabel S4.** Compound dependent parameters for negative MRMs. t_R_ retention time; DP declustering potential; EP entrance potential; CE collision energy; CXP collision cell exit potential.

| **Opioids** | **Q1** | **Q3** | **Dwell time** | **DP (V)** | **EP (V)** | **CE (V)** | **CXP(V)** |
| --- | --- | --- | --- | --- | --- | --- | --- |
| Morphin-3ßD-glucuronide-D3 | 463 | 112.9 | 5 | -80 | -10 | -35 | -12 |
| **Cannabinoids** |  |  |  |  |  |  |  |
| THC-COOH | 343.2 | 299.3 | 5 | -80 | -10 | -35 | -12 |
| THC-OH | 329.2 | 311.3 | 5 | -80 | -10 | -20 | -12 |
| **Benzodiazepines** |  |  |  |  |  |  |  |
| Aztreonam | 434.1 | 95.9 | 5 | -80 | -10 | -50 | -12 |
| **Barbiturates** |  |  |  |  |  |  |  |
| Allobarbital | 207.1 | 42 | 5 | -50 | -10 | -40 | -12 |
| Amobarbital | 225.1 | 181.2 | 5 | -50 | -10 | -18 | -12 |
| Barbital | 183 | 42 | 5 | -50 | -10 | -40 | -12 |
| Brallobarbital | 285 | 205.1 | 5 | -80 | -10 | -20 | -12 |
| Butabarbital | 211.1 | 42 | 5 | -60 | -10 | -40 | -7 |
| Butalbital | 223.1 | 42 | 5 | -65 | -10 | -40 | -7 |
| Hexobarbital | 234.9 | 42 | 5 | -45 | -10 | -45 | -12 |
| Pentobarbital | 225.1 | 138.2 | 5 | -80 | -10 | -20 | -12 |
| Phenobarbital | 231.1 | 187.9 | 5 | -35 | -10 | -15 | -12 |
| Propallylonal | 287 | 207.1 | 5 | -80 | -10 | -20 | -12 |
| Secbutabarbital | 211 | 42 | 5 | -40 | -10 | -40 | -12 |
| Secobarbital | 237.1 | 42.1 | 5 | -90 | -10 | -40 | -7 |
| **Ethanol abuse markers** |  |  |  |  |  |  |  |
| Ethyl glucuronide | 221.1 | 84.9 | 5 | -80 | -10 | -35 | -12 |
| Ethyl glucuronide-D5 IS | 226 | 84.9 | 5 | -80 | -10 | -35 | -12 |
| Ethyl sulfate | 124.8 | 96.8 | 5 | -55 | -10 | -22 | -12 |
| Ethyl sulfate-D5 IS | 130 | 79.8 | 5 | -80 | -10 | -35 | -12 |
| **Drugs** |  |  |  |  |  |  |  |
| 4-Benzamidosalicyclic acid | 256.1 | 212.1 | 5 | -80 | -10 | -35 | -12 |
| 5-(p-Methylphenyl)-phenylhydantoin | 265.1 | 116 | 5 | -80 | -10 | -35 | -12 |
| 5-Aminosalicylic acid | 152 | 108 | 5 | -80 | -10 | -20 | -12 |
| 6-Mercaptourine | 151 | 92 | 5 | -80 | -10 | -35 | -12 |
| Acetazolamide | 221 | 83 | 5 | -80 | -10 | -50 | -12 |
| Acetylsalicylic Acid | 179 | 137 | 5 | -80 | -10 | -20 | -12 |
| Adrenalone | 180.1 | 108 | 5 | -80 | -10 | -50 | -12 |
| Alprostadil | 353.2 | 113 | 5 | -80 | -10 | -50 | -12 |
| Amoxicillin | 364.1 | 206.1 | 5 | -80 | -10 | -20 | -12 |
| Atorvastatin | 557.3 | 278.2 | 5 | -80 | -10 | -50 | -12 |

| Azelaic Acid | 187.1 | 125 | 5 | -80 | -10 | -20 | -12 |
| --- | --- | --- | --- | --- | --- | --- | --- |
| Benserazid | 256.1 | 137 | 5 | -80 | -10 | -20 | -12 |
| Benzthiazide | 430 | 228.1 | 5 | -80 | -10 | -50 | -12 |
| Bezafibrate | 360.1 | 274.2 | 5 | -80 | -10 | -20 | -12 |
| Carbenoxolone | 569.4 | 469.3 | 5 | -80 | -10 | -50 | -12 |
| Chlorothiazide | 293.9 | 214 | 5 | -80 | -10 | -50 | -12 |
| Cimetidine | 251.1 | 97 | 5 | -80 | -10 | -35 | -12 |
| Cinchonine | 293.2 | 236.2 | 5 | -80 | -10 | -20 | -12 |
| Cortisone | 359.2 | 329.3 | 5 | -80 | -10 | -20 | -12 |
| Dienestrol | 265 | 93 | 5 | -90 | -10 | -42 | -12 |
| Dinoprost | 353.2 | 83 | 5 | -80 | -10 | -50 | -12 |
| Epinephrine | 182.1 | 164.1 | 5 | -80 | -10 | -20 | -12 |
| Ethosuximide | 140 | 140 | 5 | -80 | -10 | -9 | -11 |
| Furosemid | 329 | 205.1 | 5 | -80 | -10 | -35 | -12 |
| Hydrochlorothiazide | 296 | 205.1 | 5 | -80 | -10 | -35 | -12 |
| Naproxen | 229 | 169 | 5 | -60 | -10 | -20 | -12 |
| Triamcinolone | 393.2 | 345.3 | 5 | -80 | -10 | -35 | -12 |
| Valproic acid | 143 | 143 | 5 | -20 | -10 | -9 | -11 |
| **Amino acids** |  |  |  |  |  |  |  |
| Adenine | 134.1 | 107 | 5 | -80 | -10 | -35 | -12 |
| Adenosine | 266.1 | 134.1 | 5 | -80 | -10 | -50 | -12 |

**Table S5.** Evaluation of the identification of 102 analytes present in standards in urine at different concentrations. + Compound was identified. - Compound could not be identified. ^a^ Compound was identified through the combined rule despite reverse fit not optimal (> 80%). ^b^ MRM signal is positive but no acquisition of an EPI spectrum was triggered. Compound was identified through the combined rule.

| **Analytes** | **Standard solution 1** | | **Standard solution 2** | | **Standard solution 3** | |
| --- | --- | --- | --- | --- | --- | --- |
|  | **Concentration (μg/L)** | **Identification** | **Concentration (μg/L)** | **Identification** | **Concentration (μg/L)** | **Identification** |
| Amphetamine | 27.9 | + | 157 | + | 513 | + |
| BDB | 27.9 | - | 154 | - | 501 | - |
| Butylone | 28.2 | + | 157 | + | 551 | + |
| 2C-B | 23.5 | +^a^ | 141 | +^a^ | 539 | + |
| 2C-I | 24.8 | - | 153 | - | 461 | - |
| Cathinone | 27.3 | + | 166 | + | 559 | + |
| MBDB | 27 | + | 158 | + | 542 | + |
| MDA | 25.3 | - | 160 | + | 614 | + |
| MDEA | 26.4 | + | 153 | + | 565 | + |
| MDMA | 25.9 | + | 140 | + | 515 | + |
| MDPV | 27.7 | + | 158 | + | 554 | + |
| Mephedrone | 27.2 | + | 154 | + | 556 | +^b^ |
| Methamphetamine | 27.2 | +^b^ | 156 | +^b^ | 545 | +^b^ |
| Methaqualone | 27.5 | + | 147 | + | 509 | + |
| Methylone | 24.5 | + | 143 | + | 515 | + |
| Methylphenidate | 28.2 | + | 157 | + | 519 | + |
| PMA | 28.2 | + | 141 | + | 511 | + |
| Ritalinic Acid | 25.8 | +^b^ | 140 | + | 471 | + |
| Allobarbital | 78.6 | - | 446 | +^a^ | 1581 | - |
| Amobarbital | 86 | - | 411 | + | 1377 | + |
| Barbital | 75.8 | +^b^ | 493 | + | 1881 | + |
| Butalbital | 77.7 | +^a^ | 446 | + | 1740 | + |
| Hexobarbital | 84.1 | +^a^ | 461 | +^a^ | 1503 | - |
| Pentobarbital | 64.9 | - | 464 | - | 1742 | + |
| Phenobarbital | 73.5 | - | 462 | - | 1754 | - |
| Secbutabarbital | 76.6 | +^b^ | 442 | + | 1440 | + |
| Secobarbital | 74.4 | - | 443 | - | 1691 | + |
| Alprazolam | 26.6 | + | 154 | + | 525 | + |
| 7-aminoclonazepam | 23.6 | + | 141 | + | 493 | + |
| 7-aminoflunitrazepam | 23.9 | +^a^ | 145 | + | 526 | + |
| Bromazepam | 27.2 | - | 150 | + | 529 | + |
| Brotizolam | 28.2 | +^b^ | 165 | + | 586 | + |
| Chlordiazepoxide | 24.7 | +^a^ | 148 | +^b^ | 539 | + |
| Clobazam | 27.8 | + | 141 | + | 493 | + |
| Clonazepam | 25.1 | +^b^ | 146 | +^b^ | 506 | +^b^ |
| Demoxepam | 28.4 | - | 165 | + | 597 | + |
| Desalkylflurazepam | 27.6 | +^b^ | 151 | +^b^ | 540 | +^b^ |
| Desmethylflunitrazepam | 28.7 | +^b^ | 161 | + | 537 | +^b^ |
| Diazepam | 27.5 | + | 158 | + | 557 | + |
| Estazolam | 29 | + | 154 | + | 540 | + |
| Flunitrazepam | 26.6 | + | 152 | + | 560 | + |
| Flurazepam | 27 | +^a^ | 147 | +^a^ | 534 | +^a^ |
| A-hydroxyalprazolam | 25.9 | +^b^ | 149 | +^b^ | 512 | +^b^ |
| A-hydroxymidazolam | 27 | +^b^ | 154 | + | 504 | +^b^ |
| A-hydroxytriazolam | 26.2 | +^b^ | 153 | +^b^ | 509 | +^b^ |
| Lorazepam | 25.8 | + | 156 | + | 577 | +^b^ |
| Lormetazepam | 27.4 | +^b^ | 167 | +^a^ | 595 | +^a^ |
| Medazepam | 28.9 | +^b^ | 161 | +^b^ | 548 | +^b^ |
| Midazolam | 26.2 | + | 139 | + | 479 | + |
| Nitrazepam | 18.5 | +^b^ | 148 | + | 570 | + |
| Norclobazam | 28.9 | + | 164 | + | 568 | - |
| Nordiazepam | 27.2 | + | 159 | + | 575 | + |
| Oxazepam | 27.5 | - | 148 | +^b^ | 516 | +^b^ |
| Prazepam | 29.1 | - | 160 | +^a^ | 545 | +^a^ |
| Temazepam | 24.9 | + | 164 | +^b^ | 639 | +^a^ |
| Triazolam | 27.2 | +^b^ | 157 | + | 585 | +^b^ |
| Gabapentin | 26.7 | + | 160 | + | 602 | + |
| Pregabalin | 24.6 | +^b^ | 153 | + | 544 | + |
| Promethazine | 26.7 | - | 155 | - | 594 | - |
| Quetiapine | 27.9 | + | 154 | + | 546 | + |
| Benzoylecgonine | 15.5 | +^b^ | 91.4 | + | 336 | + |
| Cocaethylene | 17 | + | 96.6 | + | 346 | + |
| Cocaine | 17 | + | 95.1 | + | 347 | + |
| Norcocaine | 15.5 | + | 88.5 | + | 319 | + |
| 11-nor-9-carboxy-D9-THC | 6.89 | - | 36.6 | + | 135 | + |
| Buprenorphine | 0.566 | - | 2.98 | - | 11.2 | +^b^ |
| Codeine | 12 | - | 69.7 | +^b^ | 277 | +^b^ |
| Dihydrocodeine | 12.9 | + | 67.6 | + | 227 | + |
| EDDP | 26.1 | + | 147 | + | 526 | +^a^ |
| Fentanyl | 5.15 | + | 27.9 | + | 95.8 | + |
| Hydrocodone | 13.9 | +^b^ | 84.4 | +^b^ | 312 | +^b^ |
| Hydromorphone | 5.54 | +^b^ | 32.9 | + | 122 | +^b^ |
| Meconin | 23.3 | +^b^ | 153 | +^b^ | 521 | +^b^ |
| Meperidine | 13.5 | +^b^ | 80.1 | + | 282 | + |
| Methadone | 28.5 | + | 165 | + | 577 | + |
| 6-monoacetylmorphine | 2.56 | +^b^ | 16.8 | +^b^ | 64.1 | +^b^ |
| Morphine | 13.2 | + | 78.2 | + | 281 | + |
| Naloxone | 27.7 | + | 158 | + | 513 | + |
| Naltrexone | 27.8 | +^b^ | 153 | +^b^ | 516 | + |
| Norbuprenorphine | 0.454 | - | 3.38 | - | 12.7 | - |
| Norcodeine | 14.3 | +^a^ | 82.6 | +^a^ | 309 | +^a^ |
| Norfentanyl | 5.46 | +^b^ | 33.4 | +^b^ | 129 | + |
| Normeperidine | 14.4 | - | 81.2 | + | 287 | +^b^ |
| Nortilidine | 27.8 | +^b^ | 155 | +^b^ | 563 | +^b^ |
| O-desmethyltramadol | 27.1 | +^b^ | 157 | +^b^ | 544 | - |
| Oxycodone | 28 | + | 149 | + | 561 | + |
| Oxymorphone | 13.1 | +^b^ | 76.6 | + | 275 | + |
| Papaverine | 27.6 | + | 169 | + | 588 | + |
| Propoxyphene | 28 | +^b^ | 153 | +^b^ | 545 | +^b^ |
| Sufentanil | 28.4 | + | 155 | + | 542 | + |
| Tapentadol | 12.9 | + | 73.6 | + | 269 | + |
| Thebaine | 25.3 | + | 149 | + | 550 | + |
| Tilidine | 26.8 | +^b^ | 153 | +^b^ | 551 | +^b^ |
| Tramadol | 26.7 | + | 153 | + | 531 | + |
| Zalepon | 24.6 | + | 156 | + | 580 | + |
| Zolpidem | 28.5 | + | 164 | + | 547 | + |
| Zopiclone | 26.1 | + | 158 | + | 565 | + |
| Ketamine | 26.6 | +^b^ | 160 | + | 601 | + |
| LSD | 0.494 | +^b^ | 2.59 | +^b^ | 9.84 | + |
| Mescaline | 5.48 | + | 31 | + | 95.4 | + |
| Norketamine | 27.8 | + | 168 | + | 621 | + |
| PCP | 5.6 | +^b^ | 31.1 | + | 119 | + |

**Table S6.** Evaluation of the identification of 17 analytes present in standards in blood at different concentrations. + Compound was identified. - Compound could not be identified. ^a^ Compound was identified through the combined rule despite reverse fit not optimal (> 80%). ^b^ MRM signal is positive but no acquisition of an EPI spectrum was triggered. Compound was identified through the combined rule.

| **Analytes** | **Standard solution 1** | | **Standard solution 2** | | **Standard solution 3** | |
| --- | --- | --- | --- | --- | --- | --- |
|  | **Concentration (μg/L)** | **Identification** | **Concentration (μg/L)** | **Identification** | **Concentration (μg/L)** | **Identification** |
| Amphetamine | 10 | +^a^ | 20 | + | 50 | + |
| Metamphetamine | 10 | +^a^ | 20 | + | 50 | + |
| MDA | 10 | + | 20 | + | 50 | + |
| MDMA | 10 | + | 20 | + | 50 | + |
| THC-COOH | 2.5 | - | 5 | - | 12.5 | + |
| Cocaine | 5 | + | 10 | + | 25 | + |
| BEG | 5 | + | 10 | + | 25 | + |
| Cocaethylene | 5 | + | 10 | + | 25 | + |
| Buprenorphine | 1 | - | 2 | - | 5 | +^a^ |
| Norbuprenorphine | 1 | - | 2 | - | 5 | - |
| Codeine | 5 | + | 10 | + | 25 | + |
| Methadone | 5 | + | 10 | + | 25 | + |
| EDDP | 5 | + | 10 | + | 25 | + |
| Morphine | 5 | + | 10 | + | 25 | + |
| 6-MAM | 5 | + | 10 | + | 25 | + |
| Ketamine | 10 | + | 20 | + | 50 | + |
| Norketamine | 10 | + | 20 | + | 50 | + |

**Table S7.** Presentation of the forensic cases resulted positive including the results from the MRM-IDA-EPI screening and LC-MS/MS analysis.

| **Forensic case n. 4** | | |
| --- | --- | --- |
| **Biological matrix** | **MRM-IDA-EPI screening positivity** | **LC-MS/MS analysis** |
| Peripheral blood | Alprazolam  Nordiazepam  BEG  Codeine  Morphine | 51.79 μg/L  16.10 μg/L  284.50 μg/L  10.98 μg/L  223.80 μg/L |
| Vitreous humor | Alprazolam  Cocaine  BEG  Codeine  Morphine  6-MAM | 33.75 μg/L  47.42 μg/L  287.24 μg/L  21.61 μg/L  368.68 μg/L  48.2 μg/L |
| Synovial fluid | Alprazolam  Diazepam Nordiazepam  Cocaine  BEG  Codeine  Morphine  6-MAM | 30.78 μg/L  13.96 μg/L  17.75 μg/L  10.94 μg/L  252.22 μg/L  5.18 μg/L  58.04 μg/L  8.91 μg/L |
| **Forensic case n. 5** | | |
| **Biological matrix** | **MRM-IDA-EPI screening positivity** | **LC-MS/MS analysis** |
| Cadaveric liver | BEG  Norcocaine  THC-COOH | 5.86 ng/g  7.78 ng/g  150.04 ng/g |
| Cadaveric kidney | Cocaine  BEG  THC-COOH | 0.76 ng/g  5.26 ng/g  43.17 ng/g |
| Cadaveric spleen | Negative | Negative |
| **Forensic case n. 6** | | |
| **Biological matrix** | **MRM-IDA-EPI screening positivity** | **LC-MS/MS analysis** |
| Peripheral blood | Quetiapine  Methadone  EDDP | 62.29 μg/L  359.55 μg/L  64.12 μg/L |
| Vitreous humor | Quetiapine  Methadone  EDDP | 112.94 μg/L  370.42 μg/L  71.89 μg/L |
| **Forensic case n. 7** | | |
| **Biological matrix** | **MRM-IDA-EPI screening positivity** | **LC-MS/MS analysis** |
| Peripheral blood | 7-aminoclonazepam  Diazepam  Nordiazepam  Oxazepam  Temazepam  Lorazepam  Methadone  EDDP | 42.65 μg/L  228.90 μg/L  528.97 μg/L  16.15 μg/L  10.76 μg/L  27.99 μg/L  640. 80 μg/L  95.54 μg/L |
| **Forensic case n. 8** | | |
| **Biological matrix** | **MRM-IDA-EPI screening positivity** | **LC-MS/MS analysis** |
| Peripheral blood | 7-aminoclonazepam Alprazolam  THC-COOH | 1.09 μg/L  3.80 μg/L  6.57 μg/L |
| **Forensic case n. 9** | | |
| **Biological matrix** | **MRM-IDA-EPI screening positivity** | **LC-MS/MS analysis** |
| Urine | Lorazepam  Zolpidem | 452.82 μg/L  2640.81 μg/L |
| **Forensic case n. 10** | | |
| **Biological matrix** | **MRM-IDA-EPI screening positivity** | **LC-MS/MS analysis** |
| Urine | THC-COOH | 740.64 μg/L |
| **Forensic case n. 11** | | |
| **Biological matrix** | **MRM-IDA-EPI screening positivity** | **LC-MS/MS analysis** |
| Urine | Diazepam Nordiazepam  Oxazepam  Temazepam Lorazepam  BEG | 15.39 μg/L  660.82 μg/L  2128.76 μg/L  1724.41 μg/L  214.33 μg/L  3.91 μg/L |
| **Forensic case n. 12** | | |
| **Biological matrix** | **MRM-IDA-EPI screening positivity** | **LC-MS/MS analysis** |
| Synovial fluid | Amobarbital Pentobarbital  BEG | 1848.07 μg/L  2234.01 μg/L  6.13 μg/L |
| **Forensic case n. 13** | | |
| **Biological matrix** | **MRM-IDA-EPI screening positivity** | **LC-MS/MS analysis** |
| Synovial fluid | Alprazolam  Quetiapine  Methadone  Codeine  Morphine  6-MAM | 10.95 μg/L  15.0 μg/L  20.61 μg/L  8.32 μg/L  50.85 μg/L  **(tracce)** |
| **Forensic case n. 14** | | |
| **Biological matrix** | **MRM-IDA-EPI screening positivity** | **LC-MS/MS analysis** |
| Synovial fluid | Quetiapine  Pregabalin  Methadone | 496.60 μg/L  4350. 54 μg/L  120.49 μg/L |
| **Forensic case n. 15** | | |
| **Biological matrix** | **MRM-IDA-EPI screening positivity** | **LC-MS/MS analysis** |
| Synovial fluid | Methadone  EDDP  Codeine  Morphine | 374.63 μg/L  37.91 μg/L  9.99 μg/L  67.93 μg/L |
| **Forensic case n. 16** | | |
| **Biological matrix** | **MRM-IDA-EPI screening positivity** | **LC-MS/MS analysis** |
| Synovial fluid | Cocaine  BEG  Methadone  EDDP | 437.31 μg/L  519.47 μg/L  374.63 μg/L  37.91 μg/L |
| **Forensic case n. 17** | | |
| **Biological matrix** | **MRM-IDA-EPI screening positivity** | **LC-MS/MS analysis** |
| Cadaveric larvae | Diazepam  Nordiazepam  Oxazepam  Temazepam  Bromazepam  Codeine  Norcodeine  Morphine  Zolpidem | 58.00 ng/g  24.00 ng/g  12.00 ng/g  7.00 ng/g  3.0 ng/g  235.00 ng/g  113.00 ng/g  28.00 ng/g  18.00 ng/g |
